# A Complex Connection Between the Diversity of Human Gastric Mucin *O*-Glycans, *Helicobacter pylori* Binding, *Helicobacter* Infection and Fucosylation

**DOI:** 10.1016/j.mcpro.2022.100421

**Published:** 2022-09-29

**Authors:** Gurdeep Chahal, Médea Padra, Mattias Erhardsson, Chunsheng Jin, Macarena Quintana-Hayashi, Vignesh Venkatakrishnan, János Tamás Padra, Helen Stenbäck, Anders Thorell, Niclas G. Karlsson, Sara K. Lindén

**Affiliations:** 1 Department of Medical Biochemistry and Cell Biology, University of Gothenburg, Gothenburg, Sweden; 2 Proteomics Core Facility at Sahlgrenska Academy, Gothenburg, Sweden; 3 Department of Clinical Science at Danderyds Hospital and Department of Surgery, Karolinska Institutet, Ersta Hospital, Stockholm, Sweden; 4 Department of Life Sciences and Health, Faculty of Health Sciences, Oslo Metropolitan University, Oslo, Norway

**Keywords:** *Helicobacter pylori*, glycosylation, human stomach, inflammation, binding, BabA, blood group antigen–binding adhesion, GI, gland insoluble, GS, gland soluble, GuHCl, guanidinium chloride, HAS, human serum albumin, HRP, horseradish peroxidase, IgG, immunoglobulin G, Leb, Lewis b, MS, mass spectrometry, PVDF, polyvinylidene difluoride, RA, relative abundance, RT, retention time, SabA, sialic acid–binding adhesion, SI, surface insoluble, SLea, sialyl-Lewis a, SLex, sialyl-Lewis x, SS, surface soluble

## Abstract

*Helicobacter pylori* colonizes the stomach of half of the human population. Most *H. pylori* are located in the mucus layer, which is mainly comprised by glycosylated mucins. Using mass spectrometry, we identified 631 glycans (whereof 145 were fully characterized and the remainder assigned as compositions) on mucins isolated from 14 *Helicobacter* spp.-infected and 14 *Helicobacter* spp.-noninfected stomachs. Only six identified glycans were common to all individuals, from a total of 60 to 189 glycans in each individual. An increased number of unique glycan structures together with an increased intraindividual diversity and larger interindividual variation were identified among *O*-glycans from *Helicobacter* spp.-infected stomachs compared with noninfected stomachs. *H. pylori* strain J99, which carries the blood group antigen–binding adhesin (BabA), the sialic acid–binding adhesin (SabA), and the LacdiNAc-binding adhesin, bound both to Lewis b (Leb)-positive and Leb-negative mucins. Among Leb-positive mucins, *H. pylori* J99 binding was higher to mucins from *Helicobacter* spp.-infected individuals than noninfected individuals. Statistical correlation analysis, binding experiments with J99 wt, and J99Δ*babA*Δ*sabA* and inhibition experiments using synthetic glycoconjugates demonstrated that the differences in *H. pylori*-binding ability among these four groups were governed by BabA-dependent binding to fucosylated structures. LacdiNAc levels were lower in mucins that bound to J99 lacking BabA and SabA than in mucins that did not, suggesting that LacdiNAc did not significantly contribute to the binding. We identified 24 *O*-glycans from Leb-negative mucins that correlated well with *H. pylori* binding whereof 23 contained α1,2-linked fucosylation. The large and diverse gastric glycan library identified, including structures that correlated with *H. pylori* binding, could be used to select glycodeterminants to experimentally investigate further for their importance in host–pathogen interactions and as candidates to develop glycan-based therapies.

## Highlights

• Gastric mucins contain over 650 *O*-glycans whereof six are common to all individuals.
• *Helicobacter* spp. infection and fucosylation increase *O*-glycan diversity.
• *Helicobacter* spp. infection and fucosylation govern *H. pylori* binding.
• Novel glycans and factors such as steric hindrance may affect binding.

## Graphical Abstract

**Figure:**
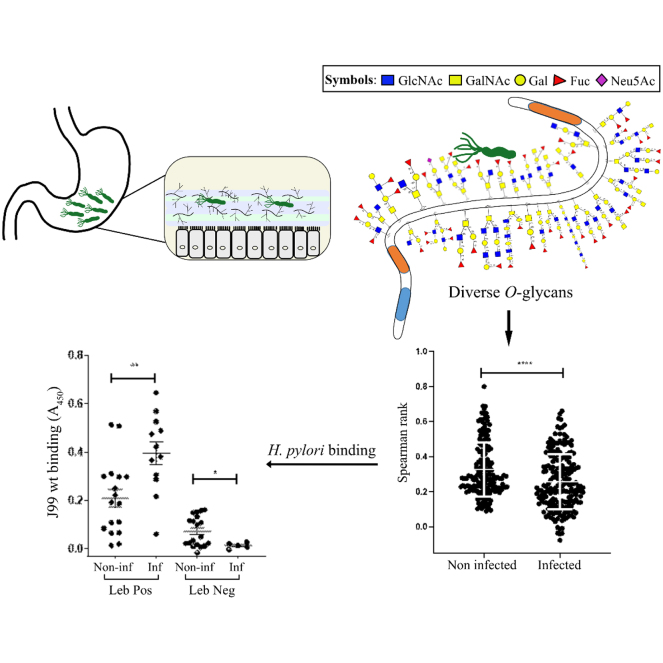

## In Brief

*Helicobacter pylori* colonizes the gastric mucus layer of half the world population. Mucin glycans in this mucus layer govern pathogen virulence, growth, and adhesion. The 650 *O*-glycan structures identified in this study represent a reliable database of structures that are of putative importance for host–pathogen interactions. We further showed that both the enormous gastric *O*-glycan diversity and avidity for *H. pylori* are governed by fucosylation and infection-associated *O*-glycans.

*Helicobacter pylori* is a microaerophilic, gram-negative, and spiral-shaped bacterium found in the stomach of approximately half of the world’s human population (1, 2). The majority of infected individuals carry and transmit *H. pylori* without any symptoms of disease (3, 4). However, *H. pylori* infection causes chronic active gastritis that may develop into more severe diseases, such as peptic ulceration (10–20%) or stomach adenocarcinoma (0.5–2%) (5, 6). *H. pylori* can be found both in the mucus layer and in contact with the epithelium (7). *H. pylori*-infected rhesus monkeys and human children secreting mucins with less *H. pylori*-binding capacity develop higher *H. pylori* density infections and more severe gastritis (7, 8). Mucins can act as steric hindrance and releasable decoys for *H. pylori* binding to the gastric epithelial surface (9). Furthermore, mice lacking Muc1 and Muc5AC have a higher *H. pylori* colonization density than mice expressing these mucins (10). Together, these results support that the ability of mucins to bind and disseminate *H. pylori* decrease the number of *H. pylori* in contact with the epithelium.

The highly glycosylated MUC5AC and MUC6 mucins are main components of the gastric mucus layer (6, 11). MUC5AC is produced by the surface epithelium of the stomach, whereas MUC6 is expressed in mucous neck cells and principal cells of the corpus and in pyloric glands of the antrum (12, 13, 14, 15). High diversity has previously been identified among mucin glycans from gastric tissues of various health status: we previously identified 258 glycan structures on mucins from three macroscopically normal and seven tumor-affected stomachs (16). Another study found 88 structures among mucins from gastric juice or tissue from ten healthy individuals, 25 asymptomatic *H. pylori*-infected individuals, and five infected individuals with intestinal metaplasia (17), with overlap between the structures identified in these two studies. In each individual, 34 to 103 *O*-glycans containing 2 to 13 monosaccharides were identified (16). Core 2 *O*-glycans are a dominant feature among gastric glycosylation, and the majority of the *O*-glycans are neutral and fucosylated (16, 17). The diversity of *O*-glycans include blood group–type ABH and Lewis (Le) epitopes, as well as terminal sialylation, α1,4-GlcNAc and GalNAcβ1–4GlcNAc (LacdiNAc)-like structures (16, 17).

*H. pylori* and the closely related *Helicobacter suis* interact with mucins *via* the mucin glycans (18, 19). *H. pylori* adheres to mucins *via* the blood group antigen–binding adhesin (BabA) that binds to Lewis b (Leb) and related fucosylated structures and the sialic acid–binding adhesin (SabA) that binds mainly to sialyl-Lewis x (SLex) and sialyl-Lewis a (SLea) (5, 6, 20). The *H. pylori* LabA has also been suggested to bind to *O*-glycans carrying a LacdiNAc motif (21, 22), but the circumstances for how to induce this binding still needs to be explored (23). A time-dependent decrease in the level of Leb and an increase of the levels of SLex/a in the gastric mucosa in rhesus monkeys experimentally infected with *H. pylori* have been demonstrated using immunohistochemistry (7). Moreover, SLea and Leb were expressed in fewer surface mucous cells after *H. pylori* eradication compared with before eradication in human stomachs (24). In addition, mucins from gastric tumors and intestinal metaplasia contained a higher proportion of negatively charged glycans, especially sulfated glycans compared with mucins from nonmalignant and noninfected stomachs, as demonstrated by mass spectrometry (MS) (16, 17). A recent study demonstrated that the gastric mucin glycosylation differed between experimentally *H. suis*-infected pigs and -noninfected controls, and that gastric mucins from *Helicobacter* spp.-infected humans and pigs had a decreased ability to bind and inhibit *H. suis* growth as compared with noninfected controls (25). Furthermore, *H. pylori* infection–related changes that affect *H. pylori* adhesion have been demonstrated in rhesus monkeys (7). However, the human gastric glycan environment and its effects on *H. pylori* during infection are poorly understood.

The aims of this study were to characterize the mucin glycosylation of *H. pylori*-infected and *H. pylori*-noninfected individuals and to investigate the effect of specific histo-blood group–dependent antigens *versus* infection- and inflammation-related changes in gastric glycosylation on *H. pylori* binding to gastric mucins. This was accomplished by characterizing gastric samples obtained by sleeve gastrectomy from obese patients, and from these selecting four groups: Leb-positive and Leb-negative samples, with and without gastritis. The gastric mucins were characterized for apo-protein type, glycosylation and *H. pylori* binding, and the relation between carbohydrate epitopes, *H. pylori* binding, gastritis, and *Helicobacter* spp. infection status was investigated.

## Experimental Procedures

### Human Samples

Human macroscopically normal gastric samples (antrum) were obtained from 100 patients with severe obesity in conjunction with vertical sleeve gastrectomy after giving written informed consent (Ersta Hospital, Sweden) during 2015 and 2016. The study protocol was approved by the regional ethics board (Regionala etikprövningsnämnden i Göteborg, Dnr 753-14). Tissue for mucin extraction was immediately frozen and stored at −80 °C, and tissue for histology was fixed in 4% phosphate-buffered formaldehyde for 24 h and then stored in 70% ethanol until processing. Patient background information was self-reported in a presurgery questionnaire.

### Histological Methods

The gastric inflammation level was scored blinded on H&E-stained tissue sections based on inflammatory cell infiltration and lymphoid follicles. Tissue sections were stained for the Leb antigen using an anti-Leb antibody (clone LE2; Biotest) as previously described (8). Representative images of immunohistochemistry for Leb on human gastric tissue sections and scores can be seen in supplemental Fig. S1.

### FISH

Five micrometer human gastric tissue sections were deparaffinized and dehydrated in sequential washes of 50%, 80%, and 95% ethyl alcohol. After air drying, a hybridization cocktail containing 40% formamide, 0.1% SDS, 0.9 M NaCl, 20 mM Tris (pH 7.4), nuclease-free water, and 10 ng/μl fluorescently labeled probes (EUB338 probe: Cy3.5 5′-GCTGCCTCCCGTAGGAGT-3′ and Helicobacter-specific probe: Alexa488 5′-TCTCAGGCCGGATACCCGTCATAGCCT-3′; Eurofins) were added to the sections, and they were incubated in a humidified chamber at 37 °C overnight. The slides were washed in wash buffer containing 0.9 M NaCl and 25 mM Tris (pH 7.4) at 50 °C for 20 min. They were dipped in room temperature distilled water very briefly, dried at room temperature, and mounted with ProLong Gold Antifade Mountant containing 4′,6-diamidino-2-phenylindole (Life Technologies; P36935).

### Isolation and Characterization of Human Gastric Mucins

The frozen tissue pieces from antrum were drenched in 10 mM sodium phosphate buffer (pH 6.5) containing 0.1 mM PMSF. The surface mucus was separated from the remainder of the tissue by gentle scraping of the tissue with a glass microscope slide and gland material by subsequent harsher scraping. The scrapings were placed into five volumes of extraction buffer (6 M guanidinium chloride [GuHCl], 5 mM EDTA, 10 mM sodium phosphate buffer, pH 6.5) containing 0.1 M PMSF, dispersed with a Dounce homogenizer, and stirred slowly at 4 °C overnight. The insoluble material was removed by centrifugation at 23,000*g* for 50 min at 4 °C (Beckman JA-30 rotor). The supernatant was saved, and the pellet was re-extracted twice with 10 ml extraction buffer each, with the supernatants pooled together. The pooled supernatants corresponded to the GuHCl-soluble mucins. The final pellet was reduced with 10 ml of 10 mM DTT in reduction buffer (6 M GuHCl, 5 mM EDTA, 0.1 M Tris–HCl buffer, pH 8.0) for 5 h at 37 °C, followed by alkylation with 25 mM iodoacetamide overnight in the dark at room temperature, and then centrifuged as aforementioned. The supernatant corresponds to the insoluble mucins. The insoluble and soluble samples were dialyzed against ten volumes of extraction buffer. The samples were centrifuged at 40,000*g* for 90 h at 15 °C in a cesium chloride density gradient with a starting density of 1.39 g/ml. The fractions were collected from the bottom of the tubes.

### Microtiter-Based Assays for Carbohydrate, MUC5AC, MUC6, Leb, and Lea Detection

Mucin samples were diluted in 0.5 M GuHCl and coated on 96-well PolySorp flat bottom plates (Thermo Fisher Scientific) overnight at 4 °C. Carbohydrate detection was carried out by periodate oxidation and detection with biotin hydrazide, as previously described (25). For mucin apoprotein detection, samples were reduced with 2 mM DTT in reduction buffer (6 M GuHCl, 5 mM EDTA, 0.1 M Tris–HCl buffer, pH 8.0) for 1 h at 37 °C, followed by alkylation with 5 mM iodoacetamide for 1 h in the dark at room temperature. For all ELISAs (26), plates were washed three times with washing buffer (PBS and 0.05% Tween-20) and blocked with 1% blocking reagent for ELISA (Roche) containing 0.05% Tween-20 (blocking buffer) for 1 h. After discarding the blocking buffer, the wells to be analyzed for MUC5AC content were incubated with the Lum5-1 antiserum (5) diluted 1:1000 in blocking buffer for 1 h, washed with washing buffer followed by incubation with horseradish peroxidase (HRP)–conjugated anti-rabbit immunoglobulin G (IgG) diluted 1:10,000. The wells to be analyzed for MUC6 content were incubated with the Lum6-3 antiserum (5) diluted 1:300 in blocking buffer for 1 h, washed with washing buffer followed by incubation with HRP-conjugated anti-rabbit IgG diluted 1:3000. The wells to be analyzed for Leb content were incubated with the Seraclone anti-Leb antibody (clone LE2), recognizes Leb, ALeb, and Bleb (27) diluted 1:1600 in blocking buffer for 1 h, washed with washing buffer followed by incubation with HRP-conjugated antimouse immunoglobulin M diluted 1:10,000. The wells to be analyzed for Lea content were incubated with the Seraclone Anti-Lea (LE1) antibody (clone LEA2; Bio-Rad), diluted 1:5000 in blocking buffer for 1 h, washed with washing buffer followed by incubation with HRP-conjugated antimouse immunoglobulin M diluted 1:10,000 for 1 h. After washing with washing buffer, 100 μl 3,3′,5,5′-tetramethylbenzidine (Sigma–Aldrich) was added to the wells. The reaction was stopped after 10 min with 100 μl 0.5 M H_2_SO_4_, and absorbance was measured at 450 nm.

### Electrophoresis and Western Blot

Mucins were run under reducing conditions on SDS agarose/PAGE composite gels, which were casted (0.5% agarose with a 0–6% polyacrylamide gradient), run, and transferred to low-fluorescent polyvinylidene difluoride (PVDF) membranes as previously described (28). The PVDF membrane was washed 10 min in water and then immersed in Li-Cor Intercept blocking buffer for 1 h. The LUM5:1 (diluted 1:1000) or LE2 (diluted 1:100) antibodies were added to the membrane and left overnight at 4 °C. The membranes were washed five times for 5 min in PBS containing 0.5% Tween-20 and incubated with IR800-anti-rabbit or IR800 antimouse diluted 1:10,000 Li-Cor Intercept blocking buffer. After 30 min, the membranes were again washed five times for 5 min in PBS containing 0.5% Tween-20 and scanned on an Odyssey CLx reader (Li-Cor) at 800 nm.

### PCR for Detection of *Helicobacter* spp. in Human Gastric Samples

DNA isolated from cultured *H. pylori* was used as positive control, and 4 M GuHCl was used as a negative control. The DNA from the samples and controls was purified with the QIAamp DNA Mini (Qiagen) kit according to the manufacturer's instructions. Primers (Table 1) were synthesized by Eurofins Genomics. The PCR was performed in 20 μl reaction volumes consisting of a final concentration of 1× Invitrogen Platinum Hot Start PCR Master Mix (2×) (Thermo Fisher Scientific), 0.5 pmol/μl forward primer, 0.5 pmol/μl reverse primer, and 5 ng/μl template DNA. The amplification program consisted of 94 °C for 5 min, 35 cycles (94 °C for 30 s, annealing at 60 °C [*glmM*, *ureC*, *Hcom*]/55 °C [*hspA*] for 30 s, extension at 72 °C for 30 s) followed by a final extension at 72 °C for 7 min. Amplification was carried out using an Applied Biosystems 2720 Thermal Cycler (Thermo Fisher Scientific). The PCR products were separated using a 2% agarose gel, stained with ethidium bromide, and visualized under a UV light.

**Table 1:** PCR primer sequences

Gene	Primer sequence (5′->3′)	Species	Product size (bp)	Source
*Hcom*	Forward: GTA AAG GCT CAC CAA GGC TAT Reverse: CCA CCT ACC TCT CCC ACA CTC	*H*. spp.	389	(42)
*ureC*	Forward: AAG CTT TTA GGG GTG TTA GGG GTT T Reverse: CTG CTT GCT TTC TAA CAC TAA CGC	*H. pylori*	294	(43)
*glmM*	Forward: TCT AAA AAC GCC CTT TCT TCT CA Reverse: ATT CGC TCA CAA ACT TAT CCC C	*H. pylori*	130	(44)
*hspA*	Forward: ACA GCA AGA TTC ATG CTC TT Reverse: CAG AAA TCG TTT TAG ACG GCA	*H. pylori*	134	(45)

### *O*-Glycan Analysis by LC–MS

Isolated gastric mucins from the four mucin origins (surface soluble [SS], gland soluble [GS], surface insoluble [SI], and gland insoluble [GI]) were characterized by LC–MS and MS/MS and were dot-blotted to PVDF membrane (Immobilon P; Millipore). The membrane was stained with 0.125% Alcian blue in 25% ethanol and 10% acetic acid (HAc) for 60 min. The membrane was then destained with methanol. *O*-glycans were released from dots by reductive β-elimination using 0.5 M NaBH_4_ in 50 mM NaOH at 50 °C overnight. The samples were desalted using cation exchange resin (AG 50W-X8) packed onto a ZipTip C18 tip. After drying in a SpeedVac, additional methanol was added to remove residual borate by evaporation. The *O*-glycans were resuspended in 10 μl of H_2_O. About 3 μl of each sample was applied to LC–MS/MS analysis. The released glycans were separated on a column (10 cm × 250 μm) packed in-house with 5 μm porous graphite particles (Hypercarb, Thermo Scientific). The glycans were eluted with a linear gradient (0–40% acetonitrile) in 10 mM ammonium bicarbonate over 40 min with a flow rate of 10 μl/min. The samples were analyzed in negative-ion mode on an LTQ linear ion trap mass spectrometer (Thermo Electron), with an IonMax standard electrospray ionization source equipped with a stainless steel needle kept at −3.5 kV. Compressed air was used as nebulizer gas. The heated capillary was kept at 270 °C, and the capillary voltage was −50 kV. Full scan (*m/z* 380–2000, two microscan, maximum 100 ms, and target value of 30,000) was performed, followed by data-dependent MS^2^ scans (two microscans, maximum 100 ms, and target value of 10,000) with normalized collision energy of 35%, isolation window of 2.5 units, activation of q = 0.25, and activation time of 30 ms. The threshold for MS^2^ was set to 300 counts. Data acquisition and processing were conducted with the Xcalibur software (version 2.0.7) (Thermo Electron). Glycans were identified from their MS/MS spectra by manual annotation and validated by available structures stored in UniCarb-DB database (2019-06 version) (29). For structural annotation, some assumptions were made in this study. Monosaccharides in the reducing end were assumed as GalNAcol for HexNAcol for structures recognized as common *O*-linked core structures. GalNAc was used for HexNAc when identified in blood group A and LacdiNAc sequences, otherwise HexNAc was assumed to be GlcNAc; hex was interpreted as Gal residues. The presence of core 1 to 4 has been reported in gastric tissue (30, 31, 32). In this study, the sequence of Hex-HexNAcol and retention time (RT) shorter than 8 min on porous graphite carbon column was assumed to be core 1 disaccharide, Hex-(HexNAc-)HexNAcol core 2 trisaccharide, HexNAc-HexNAcol core 3 or 5 disaccharide, with core 3 having shorter RT on a porous graphite carbon column (33), and HexNAc-(HexNAc-)HexNAcol as core 4 trisaccharide. Core 5 structures (isomeric to core 3) were assumed to be present only as disaccharidesaccharides and trisaccharidesaccharides and not extended further, and they were validated with RT compared with standards obtained from our previous studies (34, 35). *O*-glycans with linear cores (core 1, 3, and 5) were distinguished from branched cores (core 2 and 4) based on the presence of [M – H]^−^-223 Da and [M – H]^−^-C_3_H_8_O_4_ (equivalent to 108 Da) in MS/MS of structures with linear core (33, 36). Other sequences not containing the reported gastric *O*-linked cores were annotated as peeling products. The biosynthesis of *O*-glycans was assumed to follow the classical pathways. Chain elongation was expected to be mediated by the addition of *N*-acetyllactosamine units (Hex-HexNAc or Galβ1–4GlcNAcβ1–3). These *N*-acetyllactosamine units were either used as terminal or capped with terminal epitopes, which were corresponding to sialylation, blood group ABH, Lewis a/x, Lewis b/y, Gal-GlcNAc (for uncharacterized chain type), and LacdiNAc. Terminal HexNAc was assumed to be αGlcNAc, since distal β1,3GlcNAc residues were usually capped with Gal residues as result of highly active galactosyltransferases. Diagnostic fragmentation ions for *O*-glycans (37) were used for *de novo* sequencing. Annotated structures were submitted to the UniCarb-DR repository containing aforementioned biosynthetic and mass spectrometric evidences and assumptions. In the SNFG cartoons in Results section, the linkage information is not projected or assigned in order to save space. For comparison of glycan abundances between samples, individual glycan structures were quantified relative to the total content by integration of the extracted ion chromatogram peak area. The area under the curve of each structure was normalized to the total area under the curve and expressed as a percentage. The peak area was generated from MS raw files by Progenesis QI (Nonlinear Dynamics). A structure was considered present in a sample if having a relative abundance (RA) above 0.05%.

### *H. pylori* Culture Conditions

*H. pylori* strains J99 wt and J99Δ*babA*Δ*sabA* (kindly provided by Prof Thomas Borén, Umeå University, Sweden) were cultured on *Brucella* agar (*Brucella* Medium Base; Oxoid) containing 10% defibrinated horse blood (Thermo Fisher Scientific), 1% Iso Vitox (Oxoid), 4 mg/l amphotericin B, 10 mg/l vancomycin, and 5 mg/l trimethoprim in a microaerobic environment generated by a CampyGen gas generating sachet (Oxoid) at 37 °C.

### *H. pylori* Binding to Purified Mucins and Glycoconjugates

*H. pylori* were grown on agar plates for 48 to 72 h and harvested in PBS. The bacteria were centrifuged at 2500*g* for 3 min and then resuspended in blocking buffer containing 0.05% Tween-20. Mucin samples were diluted to 4 μg/ml in 0.5 M GuHCl and coated on 96-well PolySorp plates overnight at 4 °C. The plates were washed three times with washing buffer, and the wells were blocked for 1 h with blocking buffer. After discarding the blocking buffer, bacteria with an absorbance of 0.1 at 600 nm were diluted 1:10 in blocking buffer containing 0.05% Tween-20 and added to the plates, which then were incubated at 37 °C and 120 rpm for 2 h. For the binding inhibition assay, *H. pylori* J99 wt was incubated with 0.05 mg/ml of Leb-human serum albumin (HSA) and sLex-acetyl-phenylenediamine-HSA (IsoSep) for 20 min at room temperature prior to the incubation with mucins, and 0.05 mg/ml of HSA was included in the blocking buffer in all steps in all wells. The plates were washed three times, which was repeated between every subsequent incubation step. The plates were incubated for 1 h at room temperature with rabbit anti-*H. pylori* serum diluted 1:1000 in blocking buffer containing 0.05% Tween-20, and then another hour with HRP-conjugated donkey anti-rabbit IgG diluted 1:10,000 in blocking buffer containing 0.05% Tween-20. 3,3′,5,5′-Tetramethylbenzidine substrate (100 μl) was added, and the plates were incubated for 15 min at room temperature. The reaction was stopped by adding an equivalent amount of 0.5 M H_2_SO_4_, and the plates were read in a microplate reader at 450 nm. Control wells without mucin coating but with all bacterial strains from the aforementioned protocol were included in all experiments. The signal in control wells ranged from 0.09 to 0.15. Data in the figures are shown after subtracting this background signal. For the large binding experiments comparing the binding levels to all patient samples, samples were run with four technical replicates, and therefore, ten microtiter plates were needed for each of the J99 wt and J99Δ*babA*Δ*sabA* binding assays. To ensure difference in binding level reflected the binding ability of the mucin, two normalizations were performed. First, to ensure that the amount of mucin that was coated was the same in the wells, a glycan assay was run in parallel to the *H. pylori* binding assay. We aimed for a glycan value of 20,000, and values of 15,000 to 25,000 were accepted. The raw data were normalized to the glycan value. Each plate also contained a positive control (four technical replicates), and to allow comparison of data from different plates, the data were normalized to the mean signal of these four replicates. The variation in signal amplitude of the positive control between plates was in general less than 10% before normalization.

### Experimental Design and Statistical Rationale

**Fig. 1:**
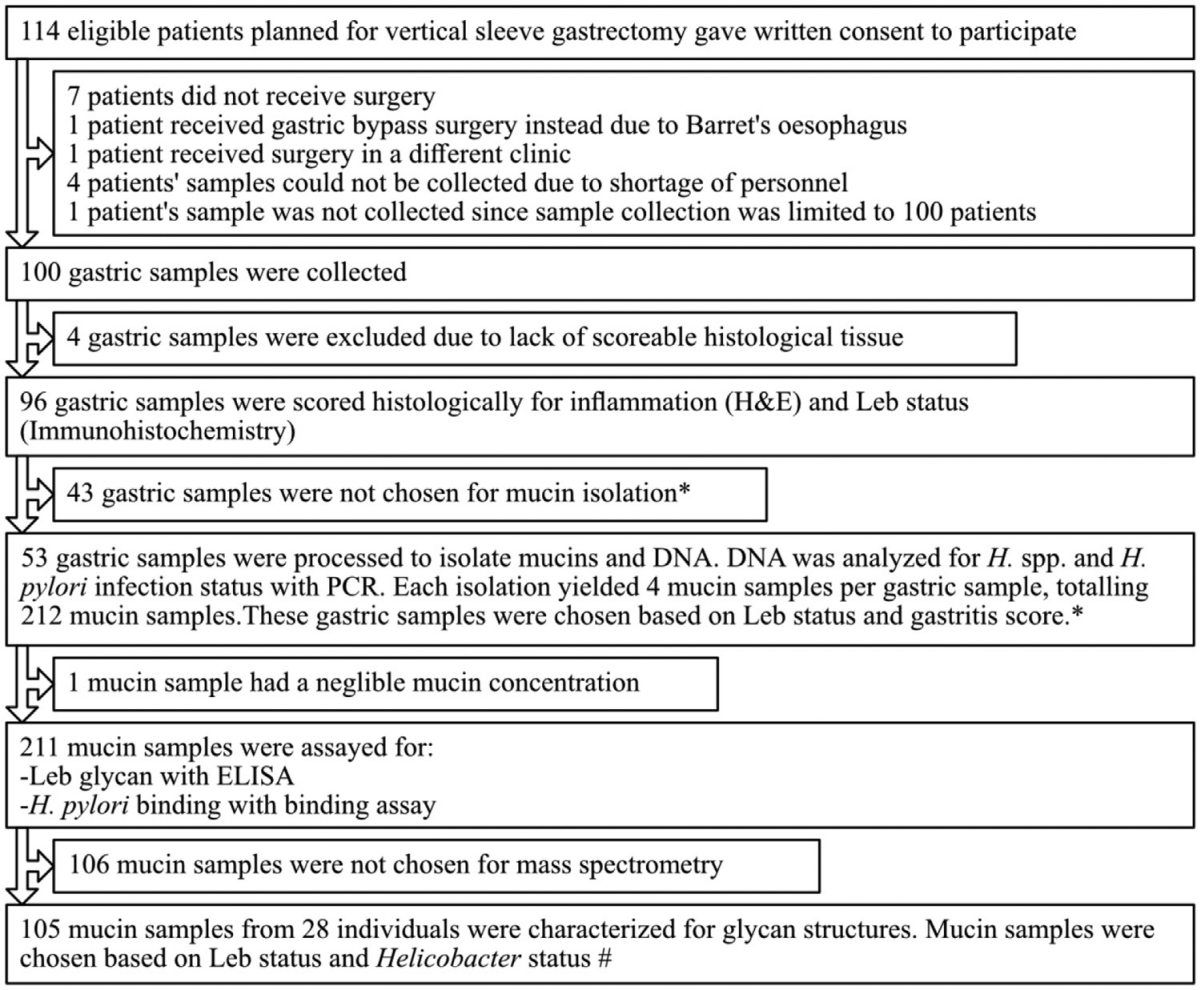
**Patient inclusion to mucin sample flowchart.** ∗Selection criteria: samples were chosen to obtain four groups (Leb positive, gastritis score <1; Leb positive, gastritis score >2; Leb negative, gastritis score <1; and Leb negative, gastritis score >2). Because of that, the majority of the population is Leb positive, the limiting sample group was Leb negative, gastritis score >2 group and samples in the Leb positive, gastritis score >2 group were selected to obtain similar gastritis scores as those in the Leb negative, gastritis score >2 group. Apart from these criteria, the samples were randomly selected within the groups. #Selection criteria: samples were chosen to obtain four groups (Leb positive, *Helicobacter* spp. infected; Leb positive, *Helicobacter* spp. negative >2; Leb negative, *Helicobacter* spp. infected; and Leb negative, *Helicobacter* spp. negative). The limiting sample group was the Leb-negative *Helicobacter* spp.-positive samples, and all samples within that group were used. Six mucin samples contained too little material to perform mass spectrometry, leading to that only three mucin sources were analyzed from these individuals. Leb, Lewis b.

The four patient groups (Leb-positive and Leb-negative individuals with and without *Helicobacter* spp. infection) consisted of 5 to 19 individuals in each group. The number of patients in the Leb-negative *Helicobacter* spp. positive group was low; because of that, there were few such individuals among the 96 samples collected (see Fig. 1 for patient sample collection flowchart). We isolated mucins from four sources from all individuals, although in one case, the amount of mucin in one of the sources was too low to perform further analysis on, and in six cases, the amount of mucin was too low to perform MS analysis; therefore, only three mucin sources were used from those individuals. Statistical analyses were performed using the GraphPad Prism 7.00 software (Dotmatics). D'Agostino–Pearson omnibus normality tests were performed to determine datasets to be analyzed with either Mann–Whitney *U* test or with Student’s *t* test. These tests were used to analyze differences between infected and noninfected groups where the data were presented per individual. Two-way ANOVA was performed to analyze the differences between infected and noninfected groups where the data were presented for several mucin sources for each individual. Differences between more than two groups and a single control group were analyzed by one-way ANOVA followed by Dunnett's multiple comparisons test. Relations between parameters were analyzed by Pearson's correlation test and corrected for multiple comparisons using the Holm–Šídák method. The *p* value for the association between Leb and J99 wt was exempt from the Holm–Šídák adjustment; because of that, BabA has experimentally been shown to bind to Leb, both in the present study and in other studies (20, 38). We do not want to unfairly cast doubt on these experimental results just because we have made many correlation analyses with the purpose of locating candidates for further experimental analysis from a large dataset. The distribution of the Spearman's rank correlation coefficient (ρ) was used as a measure of diversity within groups. Mann–Whitney *U* tests were used to compare differences between groups, for example, if the distribution of ρ between groups was larger than within groups. Differences with *p* ≤ 0.05 were considered statistically significant. Principal component analysis was conducted on glycan RA (%) data and visualized in R (version 4.0.0; R Core Team). Hierarchical clustering was done with Cluster 3.0 (39) using Spearman rank correlation and centroid linkage. Cluster visualization was performed with Java TreeView (version 1.1.6r4) (40). To calculate the differential expression in fold change between the noninfected and infected groups for the volcano plot, RA (%) of 0.1 was used for those glycans with RA (%) = 0. The value of log2FoldChange for each glycan was computed as log2 (infected/noninfected). Glycans with *p* value of ≤0.05 were considered differentially expressed.

## Results

### Histology and Patient characteristics

Samples from 96 patients with obesity were used (see Table 2 for patient characteristics). We selected 26 samples with gastritis (gastritis scores 2–5, age 24–59, 23 females) and 27 samples with no or low gastritis (gastritis scores 0–1, age 26–60, 22 females) for further analyses and mucin isolation to obtain four groups (Leb positive, gastritis score <1; Leb positive, gastritis score >2; Leb negative, gastritis score <1; and Leb negative, gastritis score >2). Among the samples with gastritis scores ≥2, *Helicobacter* spp. were identified in 12 of 26 samples using PCR for *Hcom*, whereas among the samples with gastritis scores ≤1, 5 of 27 samples were positive for *Helicobacter* spp (Table 3). The patient who had undergone *H. pylori* eradication treatment had gastritis but no postsurgery *Helicobacter* spp. infection. Thirteen of 17 *Helicobacter* spp.-positive samples were confirmed to be *H. pylori* positive by PCR for the *H. pylori* genes *hspA*, *ureC*, and *glmM*. The remaining four samples did not form a clearly visible product with primers for these three genes and therefore remained designated as *Helicobacter* spp. In the subsequent analyses, the *Helicobacter* spp.-infected samples did not differ from the samples confirmed to be *H. pylori* infected. Hereafter, all samples are therefore referred to as *Helicobacter* spp. positive. *Helicobacter* spp. were also visualized in tissue slides from *Helicobacter* spp. PCR-positive samples using a *Helicobacter* spp.-specific FISH probe, and they had a morphology consistent with *H. pylori*, regardless of if the samples were positive for the *H. pylori*-specific PCR genes or only for *Hcom*. No non-*Helicobacter* bacteria were found with a general eubacterial probe in tissue sections from either *Helicobacter* spp.-positive or -negative samples (supplemental Fig. S2). See Table 3 for group characteristics of *Helicobacter* spp.-infected *versus*-noninfected patients.

**Table 2:** Patient characteristics for all samples collected

Patient characteristics	All samples (n = 96)	Gastritis ≤1 (n = 54)	Gastritis ≥2 (n = 42)
Female (%/number)	78%/n = 75	76%/n = 41	81%/n = 34
Age (mean/median/range)	41.1/42.0/19–64	42.6/44.0/19–64	39.3/39.5/24–59
Body mass index (kg/m^2,^, mean/median/range)	39.91/39.20/32.70–53.50	40.28/39.55/32.70–53.50	39.43/39.00/34.00–48.90
Obesity (%)	100	100	100
Healthy (apart from obesity, %/number)	31%/n = 30	30%/n = 16	33%%/n = 14
Diabetes (type 1 or 2, %/number)	10%/n = 10	7%/n = 4	14%/n = 6
Proton pump inhibitor treatment (%/number)	9%/n = 9	6%/n = 3	14%/n = 6
Gastritis (histology score 0–5, mean/median/interquartile range)	1.47/1.00/2.00	0.44/0.00/1.00	2.79/2.50/1.00
Leb (histology score 0–5, mean/median/interquartile range)	1.54/2.00/2.00	1.30/1.00/2.00	1.86/2.00/2.00^a^

Representative images for the Leb staining are shown in supplemental Fig. S1.

a The Leb scores among patients with gastritis scores ≤1 were lower than from those with gastritis scores ≥2, Mann–Whitney *U* test, *p* < 0.05. Means and medians are presented together to nuance the data where applicable. The data presented as % were analyzed with Chi-square test, but no differences were significant between groups.

**Table 3:** Patient characteristics, gastritis, and *Helicobacter* spp. status for the patients from whom mucins were isolated from (A) and for the patients from whom *O*-glycans were characterized from (B)

A	*Helicobacter* spp. negative (n = 36)	*Helicobacter* spp. positive (n = 17)
Female (%/number)	83%/n = 30	88%/n = 15
Age (mean/median/range)	41.9/43.0/24–60	43.8/45.0/28–59
Body mass index (kg/m^2,^, mean/median/range)	40.64/40.20/34.00–53.50	38.72/38.10/34.80–46.10
Healthy (apart from obesity, %/number)	28%/n = 10	41%/n = 7
Diabetes (type 1 or 2, %/number)	11%/n = 4	6%/n = 1
Proton pump inhibitor treatment (%/number)	8%/n = 3	12%/n = 2
Gastritis (histology score 0–5, mean/median/interquartile range)	1.25/0.50/2.25	2.77/3.00/3.00^a^
Lea+ (%/number)	47/%/n = 7	71%/n = 12
Leb+ (%/number)	42%/n = 15	41%/n = 7
Lea+/Leb+ (%/number)	11%/n = 4	12%/n = 2
Lea+/Leb− (%/number)	36%/n = 13	59%/n = 10
Lea−/Leb+ (%/number)	31%/n = 11	29%/n = 5
Lea−/Leb− (%/number)	22%/n = 8	0%/n = 0
Blood group O (%/number)	22%/n = 8	35%/n = 6
Blood group A (%/number)	64%/n = 23	35%/n = 6
Blood group B (%/number)	11%/n = 4	17%/n = 3
Blood group AB (%/number)	3%/n = 1	12%/n = 2

B	*Helicobacter* spp. negative (14 individuals/53 samples)	*Helicobacter* spp. positive (14 individuals/52 samples)
Female (%/number)	79%/n = 11	86%/n = 12
Age (mean/median/range)	45.9/48.0/28–57	43.1/43.5/28–59
Body mass index (kg/m^2^) (mean/range)	41.25/40.35/37.30–49.90	38.48/38.20/34.80–45.00
Healthy (apart from obesity, %/number)	36%/n = 5	36%/n = 5
Diabetes (type 1 or 2, %/number)	14%/n = 2	7%/n = 1
Protom pump inhibitor treatment (%/number)	7%/n = 1	14%/n = 2
Gastritis (histology score 0–5, mean/median/interquartile range)	0.07/0.00/0.00^a^	3.14/3.00/1.00^a^
Lea+ (%/number)	50%/n = 7	43%/n = 6
Leb+ (%/number)	50%/n = 7	64%/n = 9
Lea+/Leb+ (%/number)	14%/n = 2	7%/n = 1
Lea+/Leb&minus (%/number)	36%/n = 5	57%/n = 8
Lea−/Leb+ (%/number)	36%/n = 5	36%/n = 5
Lea−/Leb− (%/number)	14%/n = 2	0%/n = 0
Blood group O (%/number)	29%/n = 4	29%/n = 4
Blood group A (%/number)	57%/n = 8	36%/n = 5
Blood group B (%/number)	7%/n = 1	21%/n = 3
Blood group AB (%/number)	7%/n = 1	14%/n = 2

a Refers to that the gastritis scores for samples positive for *Helicobacter* spp. were higher than from negative samples, Mann–Whitney *U* test, *p* < 0.05. Means and medians are presented together to nuance the data where applicable. ABO blood group was obtained from the patient charts, whereas Leb and Lea presence on mucins were analyzed by ELISA.

### Isolation and Characterization of Human Gastric Mucins

**Fig. 2:**
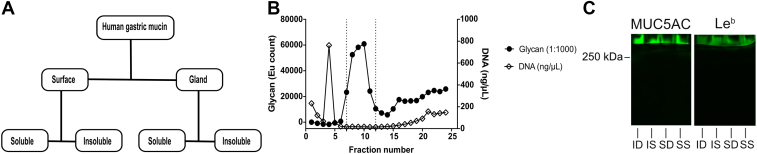
**Isolation and characterization of human gastric mucins.***A*, each specimen was separated into four types of mucin samples based on their tissue location and solubility in GuHCl. *B*, mucins isolated from these samples using isopycnic cesium chloride density gradient centrifugation (representative graph). Fractions from the density gradient were analyzed for carbohydrate (*black circle*) and DNA (absorbance at 260 nm, *white diamond*) contents. The absorbance at 260 nm that represents the DNA is present as a sharp peak in fraction 4, whereas the absorbance at 260 nm present as a slope above fraction 15 represents nonmucin proteins. The *vertical dashed lines* indicate the mucin-containing fractions that were pooled for further experiments. *C*, Western blot from an SDS-AgPAGE (0.5% agarose with a 0–6% polyacrylamide gradient) of soluble mucins from surface soluble (SS), insoluble mucins from surface (SI), soluble mucins from the glands/deeper tissue (SD), insoluble mucins from the glands/deeper tissue (ID), stained with antibodies against MUC5AC and Leb. The *line* where the mucin bands were halted represents the start of the polyacrylamide gradient, that is, the mucins remained in the part of the gel that contained only agarose. AgPAGE, agarose PAGE; GuHCl, guanidinium chloride; Leb, Lewis b.

Gastric mucins were isolated from the antral region of the human gastric mucosa. During the extraction of mucins from the tissue, the surface mucosa was mechanically separated from the deeper gland material, and thereby, MUC5AC was enriched in the surface material (supplemental Table S1). Extracted mucins were divided into soluble and insoluble groups based on their solubility in GuHCl resulting in four samples for each individual: SS, SI, GS, and GI (Fig. 2*A*). The mucins were purified from other proteins and DNA using isopycnic density gradient centrifugation, and the mucin-containing fractions were pooled for further experiments (Fig. 2*B*). This method of mucin isolation resulted in large mucin molecules, as illustrated by that antibody reactivity against both MUC5AC and Leb remained at the top of an SDS agarose/composite PAGE (0.5% agarose with a 0–6% polyacrylamide gradient), well above the 250 kD marker (Fig. 2*C*).

### Distribution and Diversity of Gastric Mucin *O*-glycans

#### About 631 Oligosaccharides Were Detected Whereof Only Six Were Common to All Individuals

**Fig. 3:**
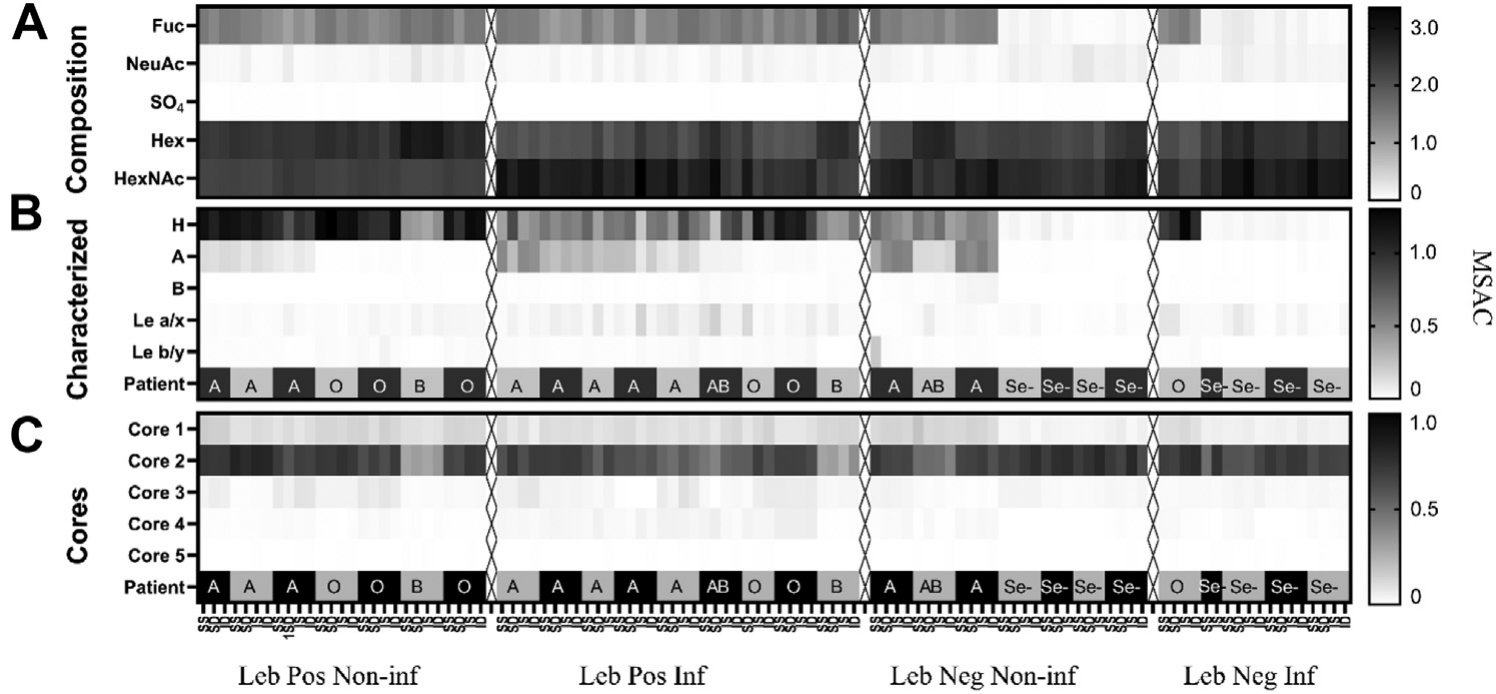
**Heatmaps visualizing the distribution and of human gastric mucin *O*-glycans from noninfected and *Helicobacter* spp.-infected stomachs.***A*, the composition of all 631 glycans. Please note that the fact that several of the blood group B and AB individuals appears to have increased levels of Hex may be due to that we were not able to assign these structures from the composition. *B*, the average number of histo-blood group motifs per glycan among the 145 fully characterized glycans. *C*, the average number of core structures among the 145 fully characterized glycans. Se^−^ denotes secretor-negative individuals based on their low amount of blood group structures identified (<0.1%). SS denotes that the glycans are from GuHCl soluble mucins from the antral surface, SI insoluble mucins from antral surface, DS soluble mucins from the antral glands, and DI insoluble mucins from the antral gland and MSAC. MSAC, MS based average composition; GuHCl, guanidinium chloride.

Mucin *O*-glycans from the four mucin origins (SS, GS, SI, and GI) from *Helicobacter* spp.-infected (n = 14) and -noninfected (n = 14) stomachs were characterized by LC–MS and MS/MS (Fig. 3 and supplemental Table S2). Samples were chosen to obtain four groups (Leb positive, *Helicobacter* spp. infected; Leb positive, *Helicobacter* spp. negative; Leb negative, *Helicobacter* spp. infected; and Leb negative, *Helicobacter* spp. negative). See Table 3 for group characteristics of *Helicobacter* spp.-infected *versus*-noninfected patients that mucin *O*-glycans were characterized for. A total of 631 glycan peaks were detected with LC/MS whereof 145 abundant glycans were fully characterized and the remainder assigned as compositions (supplemental Table S2). Fifty-six of the structures were novel, whereas the remainder have been reported previously (16, 34, 35, 41). The fully characterized glycans constituted 79.9 ± 5.4% (SD) of the total area under the peaks. Based on composition from MS intensities, neutral oligosaccharides were the dominating structures among both infected and uninfected individuals with an RA of 91.6 ± 0.5% (mean ± SEM), whereof most were fucosylated (RA: 63.2 ± 3.4%, Fig. 3*A*). The dominating core was core 2 (81 fully characterized structures) with an RA of 63.5 ± 1.4%, whereas core 1 (20 structures, RA: 9.3 ± 0.4), core 3 (12 structures, RA: 2.6 ± 0.2), and core 4 (18 structures, RA: 1.3 ± 0.1) were less prevalent (Fig. 3*C*). Core 5 disaccharide GalNAcα1–3GalNAc was sporadically detected with an abundance of less than 0.1%. The none–core-associated sialyl-Tn was detected on the average level of 1%, whereas the Tn-antigen monosaccharide was not detectable because of its early elution in the chromatogram. Fourteen structures (RA < 2%) were assigned as peeling products because of the absence of the initiating GalNAc (supplemental Table S2, characterized glycans).

Among the fully characterized structures, fucosylated structures dominated, where blood group H was the most common epitope (51 structures), followed by blood group A (28 structures, Fig. 3 and supplemental Table S2). The abundance of blood group B was low, both regarding the number of individuals in our sample set carrying this epitope, and the number of identified structures (four structures). Type 2 and type 3 were the dominating blood group–containing structures (Fig. 3). Blood group H type 1 was only positively identified in the structure Fucα1–2Galβ1–3GlcNAcβ1–3GalNAcol, and type 1 sequence without fucosylation was only positively identified in a structure assigned as a potential degradation product. The data indicate that group H type 1 structures are part of larger oligosaccharides not structurally determined in this report. In addition, using MS/MS, we also characterized fucosylation in Lea/x and Leb/y (including extension into A/B) Leb/y (Fig. 3 and supplemental Table S2). The sialylated version of Lea/x was only positively identified in one structure (Galβ1–3[NeuAcα2–3Galβ1–3(Fucα1–4)GlcNAcβ1–6]GalNAcol, supplemental Table S2). Sialylation mostly occurred linked to Gal residues (6% of total intensity), and the lack of six-linked NeuAc-specific fragmentation indicated that sialic acid was predominantly three-linked. Six-linked sialic acid was only positively identified linked to the reducing end GalNAc (six structures). In addition to terminating fucosylation and sialylation, we also detected abundant termination assigned as GlcNAcα1–4 (23 structures) and LacdiNAc (13 structures). Sulfation was low (0.1 ± 0.2%), and sulfate linked to GlcNAc (four structures) and Gal (one structure) residues was identified.

Only six structures of all characterized were present in all individuals (Table 4), albeit only at an RA of 0.1 to 0.3% in some individuals. The median RA of these six structures together was less than 15%, whereof the blood group H structure Fucα1–2Galβ1–3(Fucα1–2Galβ1–4GlcNAcβ1–6)GalNAcol made up the main part (Table 4). Another nine structures were present in more than 50% individuals and with a maximum RA of 10% or more (Table 4). We identified similar total numbers of structures from mucins from surface and glandular tissue (566 and 578, respectively, taking into account both fully characterized structures and structures assigned as compositions). Of the detected structures, 79% were common to both tissues. The RA of the structures unique to either surface or gland was low. However, a larger proportion of unique sialylated structures were present in the glands, whereas unique fucosylated structures were more abundant among the surface mucins (*p* < 0.05, supplemental Fig. S3).

**Table 4:** Common and abundant human gastric mucin *O*-glycans

A. Structures common to all individuals			
Mass	Structures	RA %, median (minimum–maximum)	SNFG
384	Galβ1–3GalNAcol	0.5 (0.23–3.7)	
513	NeuAcα2-6GalNAcol	0.3 (0.03–1.4)	
587	Galβ1–3(GlcNAcβ1–6)GalNAcol	0.4 (0.05–2.2)	
675	Galβ1–3(NeuAcα2–6)GalNAcol	0.2 (0.02–0.7)	
675	NeuAcα2–3Galβ1–3GalNAcol	0.1 (0.02–0.9)	
1041	Fucα1–2Galβ1–3(Fucα1–2Galβ1–4GlcNAcβ1–6) GalNAcol	11.2 (0.04–38.05)	

B. Additional abundant structures				
Mass	Structures	RA % (minimum–maximum)	Present in % of individuals	SNFG
749	Galβ1–3(Galβ1–4GlcNAcβ1–6)GalNAcol	0.0–52.8	96.4	
1114	Galβ1–4GlcNAcβ1–3Galβ1–3(Galβ1–4GlcNAcβ1–6) GalNAcol	0.0–39.0	96.4	
895	Fucα1–2Galβ1–3(Galβ1–4GlcNAcβ1–6)GalNAcol	0.0–17.3	92.9	
1244	Fucα1–2Galβ1–3(GalNAcα1–3(Fucα1–2)Galβ1–4GlcNAcβ1–6) GalNAcol	0.0–18.6	50.0	
530	Fucα1–2Galβ1–3GalNAcol	0.0–14.7	89.3	
790	Galβ1–3(GalNAcβ1–4GlcNAcβ1–6)GalNAcol	0.0–22.0	96.4	
1040	NeuAcα2-3Galβ1–3(Galβ1–4GlcNAcβ1–6)GalNAcol	0.0–21.4	78.6	
936	Fucα1–2Galβ1–3(GalNAcβ1–4GlcNAcβ1–6)GalNAcol	0.0–14.5	82.1	
895	Galβ1–3(Fucα1–2Galβ1–4GlcNAcβ1–6)GalNAcol	0.0–10.6	96.4	

(A) Mucin *O*-glycan structures present in all 28 individuals analyzed. (B) Structures present in ≥50% of the individuals with a maximum RA close to 10% or above. Symbol nomenclature for glycans, = SNFG, () = Fuc, () = Gal, () = GlcNAc, () = GalNAc, and () = NeuAc.

#### Larger Interindividual Variation Among *O*-Glycans From *Helicobacter*-Infected Than -Noninfected Individuals

**Fig. 4:**
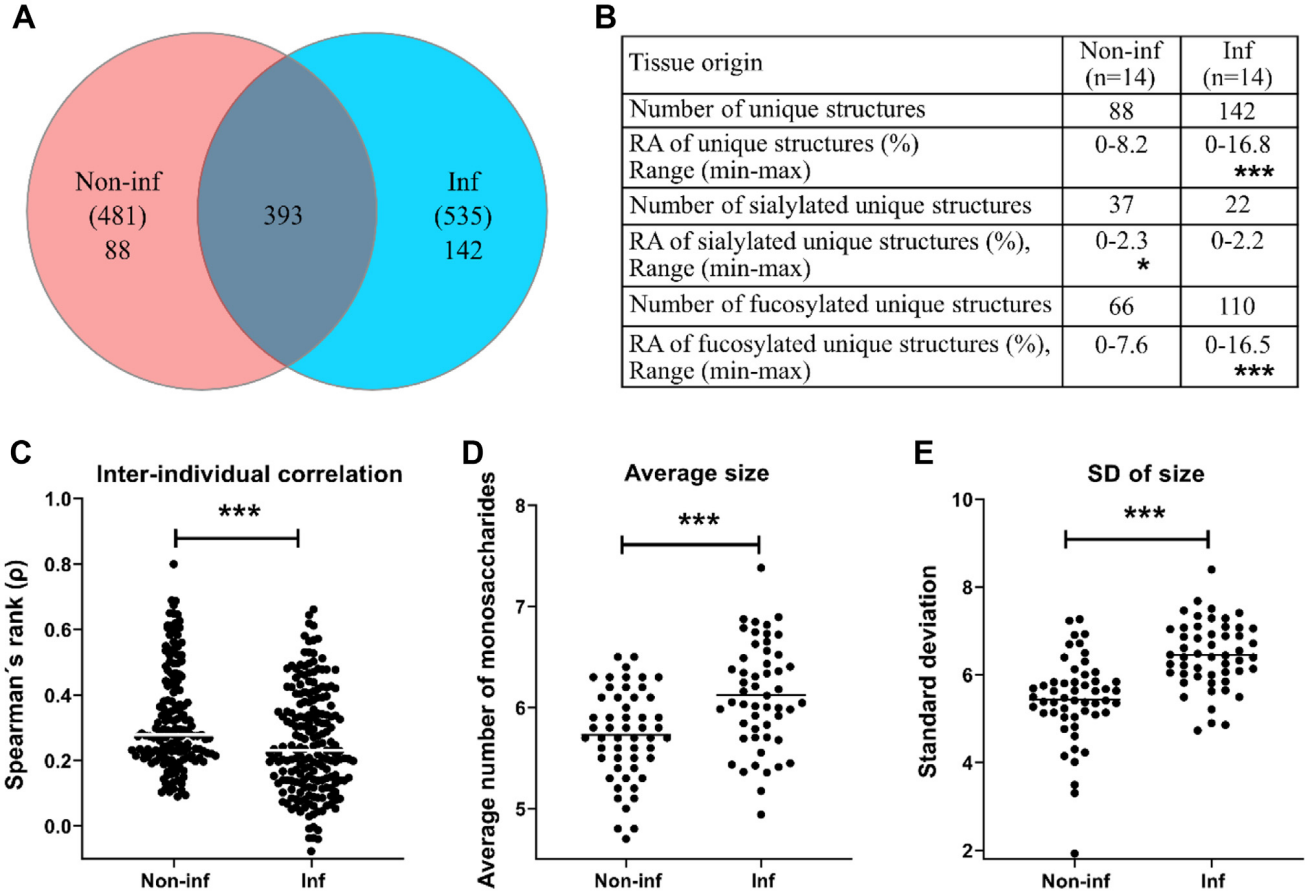
**Distribution and diversity of human gastric mucin *O*-glycans from noninfected and *Helicobacter* spp.-infected stomachs.***A*, Venn diagram of glycans on mucins from noninfected (Non-inf, n = 14) and infected (Inf, n = 14) stomachs. *B*, the relative abundances (RAs) of the glycans unique to each group are shown in the table. *Stars* indicate statistically significant higher abundance using the Mann–Whitney *U* tests. *C*, interindividual correlation in surface mucin *O*-glycan repertoire. Spearman′s rank correlation coefficients (ρ) of the RA of the gastric surface glycan structures (using the mean of the 3–4 mucin sources per patient) were compared between noninfected and infected groups using the Mann–Whitney *U* test. *D*, the average number of monosaccharides in the glycan structures and its standard deviation (3–4 mucin sources per patient). *E*, the bars represent the median, and significance was calculated using two-way ANOVA, ∗, ∗∗, and ∗∗∗ indicate *p* ≤ 0.05, <0.01, and <0.001, respectively.

Including both fully characterized structures and oligosaccharides assigned only as compositions, 535 oligosaccharides were identified among the *Helicobacter* spp.-infected individuals, and 481 oligosaccharides among the noninfected individuals, whereof 393 oligosaccharides (60%) were detected in both groups (Fig. 4*A*). Both the number and RA of uniquely identified structures were larger in the infected than in the noninfected group (Fig. 4*B*), suggesting an increased glycan diversity among infected individuals. We analyzed the correlation between the glycan profiles of each sample within groups and used the Spearman's rank correlation coefficient (ρ) as a measure of interindividual similarity. The median ρ was higher among mucin glycans from noninfected than infected individuals (Fig. 4*C*, *p* < 0.0001), demonstrating a larger interindividual diversity among glycans originating from the *Helicobacter* spp.-infected individuals compared with noninfected individuals. The average number of monosaccharides in the glycans was larger on mucins from *Helicobacter* spp.-infected individuals compared with noninfected individuals (Fig. 4*D*), which allows for an increased diversity of possible glycans that can be biosynthesized. Furthermore, the variation in glycan size within each individual was also greater among mucins from *Helicobacter* spp.-infected individuals compared with noninfected individuals, as demonstrated by the standard deviations on the intraindividual glycan size (Fig. 4*E*).

#### *O*-Glycan Repertoires Partially Cluster by Leb and Infection Status, and Fucosylation Contributes to Diversity

**Fig. 5:**
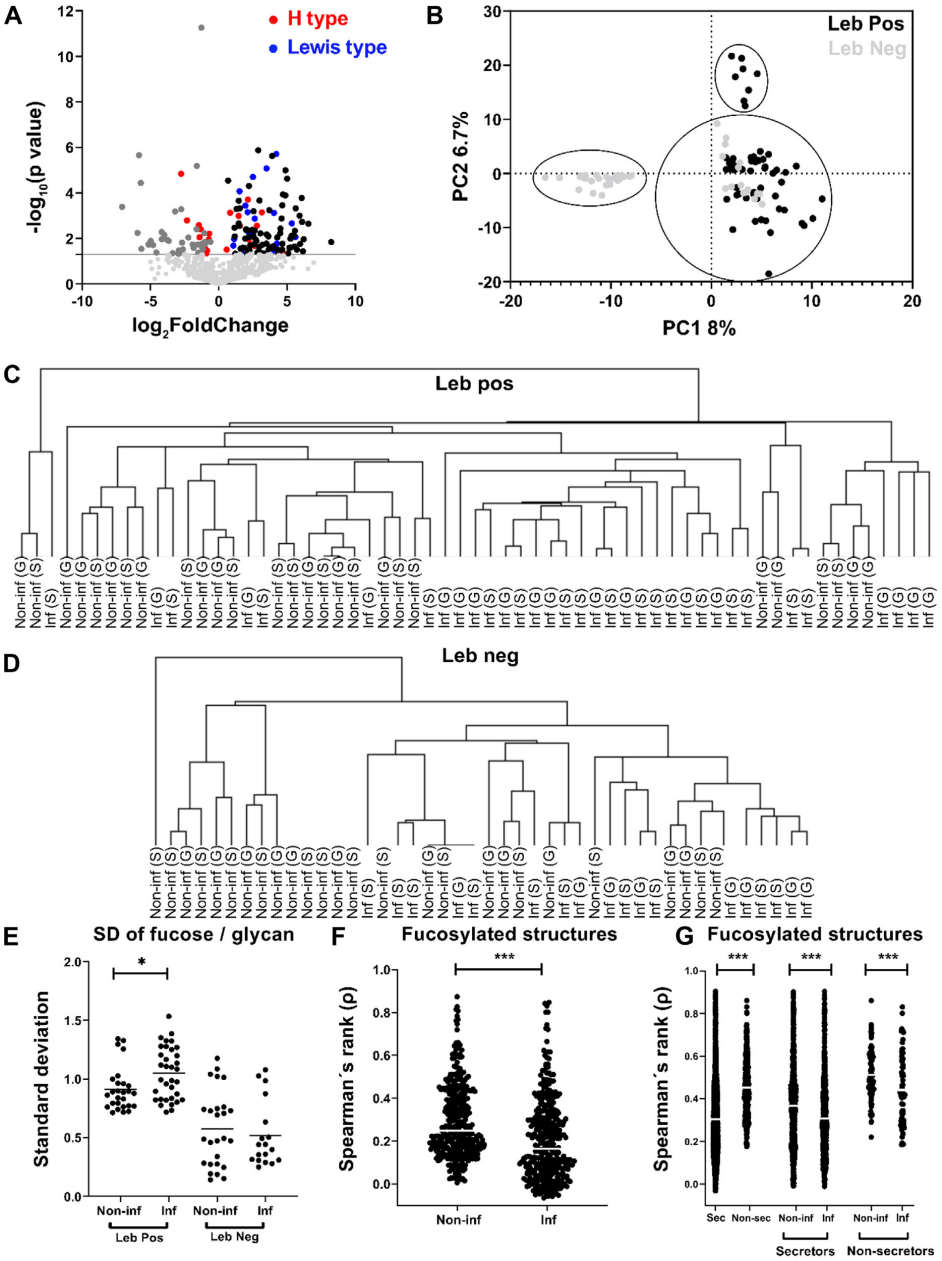
**Distribution of Lewis structures and fucosylation on mucins from individuals with and without *Helicobacter* spp. infection.***A*, volcano plot of differentially expressed gastric glycans between noninfected and *Helicobacter* spp.-infected individuals (mean of all four mucin fractions). *Dots* to the *left* of zero denote glycans expressed predominantly in noninfected individuals. *Dots* to the *right* indicate glycans more expressed in infected individuals. *Red dots* denote H-type structures, and *blue dots* denote Le-type structures (Leb/y and Lea/x). The *horizontal gray line* indicates *p* = 0.05. Below this line, the *light gray* represents glycans that were not differentially expressed. *B*, principal component analysis (PCA) based on *O*-glycan relative abundances (RAs) for all samples. Each *dot* represents a mucin source from each individual (X-variables; n = 631). The *black dots* indicate Leb positive, and *gray dots* represent Leb-negative samples based on ELISA. *C* and *D*, dendrogram after hierarchal clustering of *Lewis structures* from human mucins from surface (S, mean of SS + SI) and gland (G, mean of SD + ID) isolated from noninfected (Non-inf) and infected (Inf) stomachs. *E*, the standard deviation of the number of fucose per glycan in Leb-positive and Leb-negative samples. The *bars* represent mean, and significance was calculated using two-way ANOVA. *F*, Spearman's rank correlation coefficient (ρ) of the RA of the gastric surface fucosylated glycans. *G*, Spearman's rank correlation coefficient (ρ) of the RA of the gastric surface fucosylated glycans grouped based on secretor (sec) status. The *bars* represent the median, and significance was calculated using the Mann–Whitney *U* test. ∗ and ∗∗∗ indicate *p* < 0.05 and *p* < 0.0001, respectively. SI, surface insoluble; SS, surface soluble.

Based on the expression of blood group structures, both the noninfected and *H. pylori*-infected groups contained ten individuals with the secretor phenotype and four with the nonsecretor phenotype (Fig. 3). However, both the RA and the number of unique fucosylated structures were higher among mucins from infected than noninfected individuals (Fig. 4*B*). Among the differentially expressed structures, the Le-type structures were overrepresented among the *Helicobacter* spp.-infected individuals, whereas H type 1 to 3 structures were similarly distributed among individuals with and without *Helicobacter* spp. infection (Fig. 5*A*). In line with these results, hierarchical cluster analysis of Lewis-type structures largely clustered the samples into infected and noninfected groups within the Leb-positive and Leb-negative groups (Fig. 5, *C* and *D*). Principal component analysis clustered the sample glycan repertoires into three populations: one that was Leb positive, one that was Leb negative, and one that contained both Leb-positive and Leb-negative samples (Fig. 5*B*). The Leb-negative samples that clustered with the Leb-positive ones all carried a large proportion of H-type structures (12.7–53.3; minimum–maximum%) and were all secretor individuals based on the glycans expressed (Fig. 3), whereas the Leb-negative samples that clustered separately from the Leb-positive samples were very low in H-type structures (0.2–4.0; minimum–maximum%). The standard deviations of number of fucose per structure, used as a measure of heterogeneity of fucosylation within a sample, was larger among the Leb-positive *Helicobacter* spp.-infected individuals compared with Leb-positive noninfected individuals (Fig. 5*E*). Furthermore, the interindividual correlation for the fucosylated structures, measured as median ρ, was higher among mucins from noninfected than infected individuals (Fig. 5*F*, *p* < 0.0001). The diversity of fucosylated structures was lower among nonsecretors than secretors; however, the diversity of fucosylated glycans was higher among infected individuals both within the secretor and nonsecretor groups than among noninfected individuals. Hence, the lower correlation for fucosylated structures of the *Helicobacter* spp.-infected compared with -uninfected individuals and the increased heterogeneity of fucosylated structures within the *Helicobacter* spp.-infected individuals suggest that fucosylation contributes to the overall increased diversity in *Helicobacter* spp.-infected individuals.

### *H. pylori* Binding to Mucins

#### Among Leb-Positive Mucins, *H. pylori* Binding Was Higher to Mucins From *Helicobacter* spp.-Infected Individuals Than Noninfected individuals, Whereas Among Leb-Negative Mucins, *H. pylori* Binding Was Lower to Mucins From *Helicobacter* spp.-Infected Individuals Than Noninfected Individuals

**Fig. 6:**
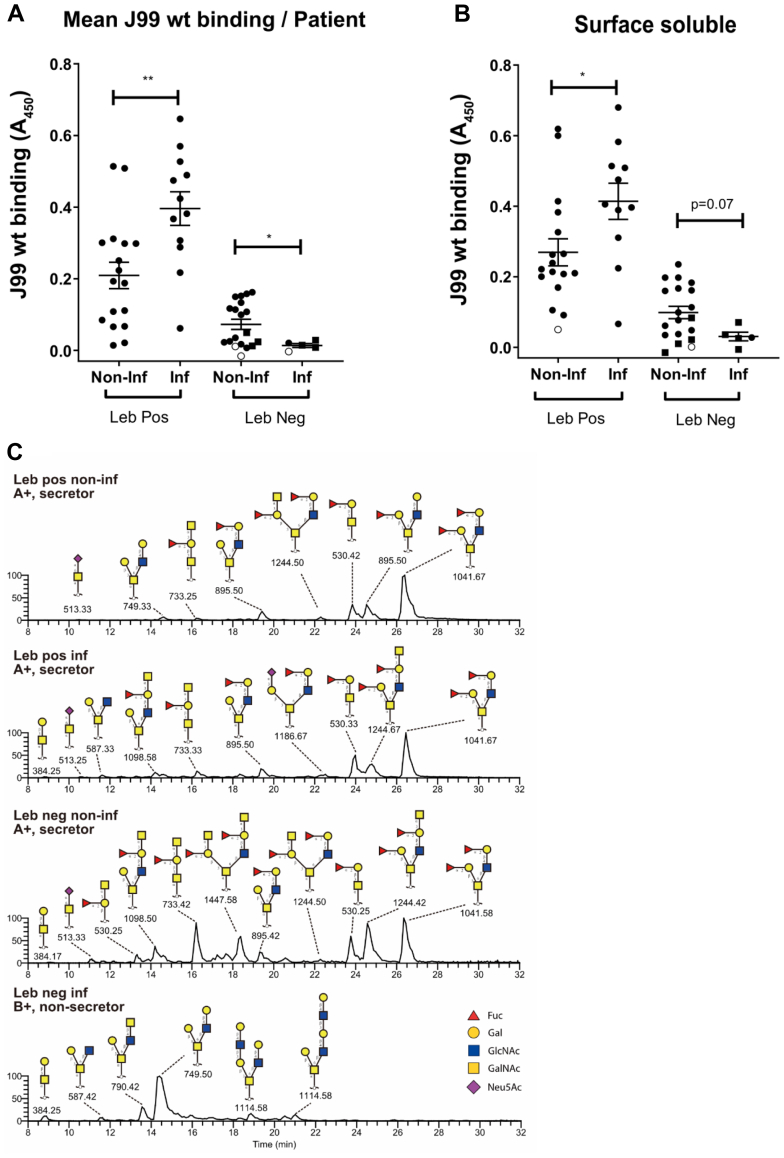
**Binding of *Helicobacter pylori* strain J99 wt to human gastric mucins isolated from *Helicobacter* spp.-infected (Inf) and -noninfected (Non-inf) individuals.***A*, the mean binding signal from the four mucin sources from each individual. *B*, the binding signal from mucins from the surface soluble mucin source from each individual. The other three mucins sources can be found in supplemental Fig. S2. *Circles* denote samples from secretors and *squares* from nonsecretors. *White symbols* denote samples in which none of the four mucin types bound statistically significantly better to J99 wt than wells with no mucins from the same microwell plate, and *black symbols* denote samples that bound J99 wt. The *bars* represent mean ± SEM, ∗*p* < 0.05 and ∗∗*p* < 0.01; *A* and *B*: Student’s *t* test. *C*, LC–MS chromatograms of *O*-glycans from the surface soluble mucin sample within each group with the highest avidity for *H. pylori* J99 wt.

Binding of *H. pylori* strain J99 wt (which carries the BabA, SabA, and LabA adhesins (23)) to Leb-positive mucins from infected (n = 12) and noninfected (n = 17) as well as Leb-negative infected (n = 5) and noninfected (n = 19) individuals was analyzed. *H. pylori* binding to mucins positive for Leb from *Helicobacter* spp.-infected individuals was higher compared with mucins from noninfected Leb-positive individuals (Fig. 6*A*, *p* < 0.01). In contrast, *H. pylori* binding to Leb-negative mucins from infected individuals was lower compared with mucins from noninfected individuals (Fig. 6*A*, *p* < 0.05). Similar results were obtained for all mucin sources (Fig. 6*B* and supplemental Fig. S4). The LC–MS chromatograms of the SS mucin sample within each group with the highest level of binding for *H. pylori* (Fig. 6*B*) has been included in Figure 6*C*.

**Fig. 7:**
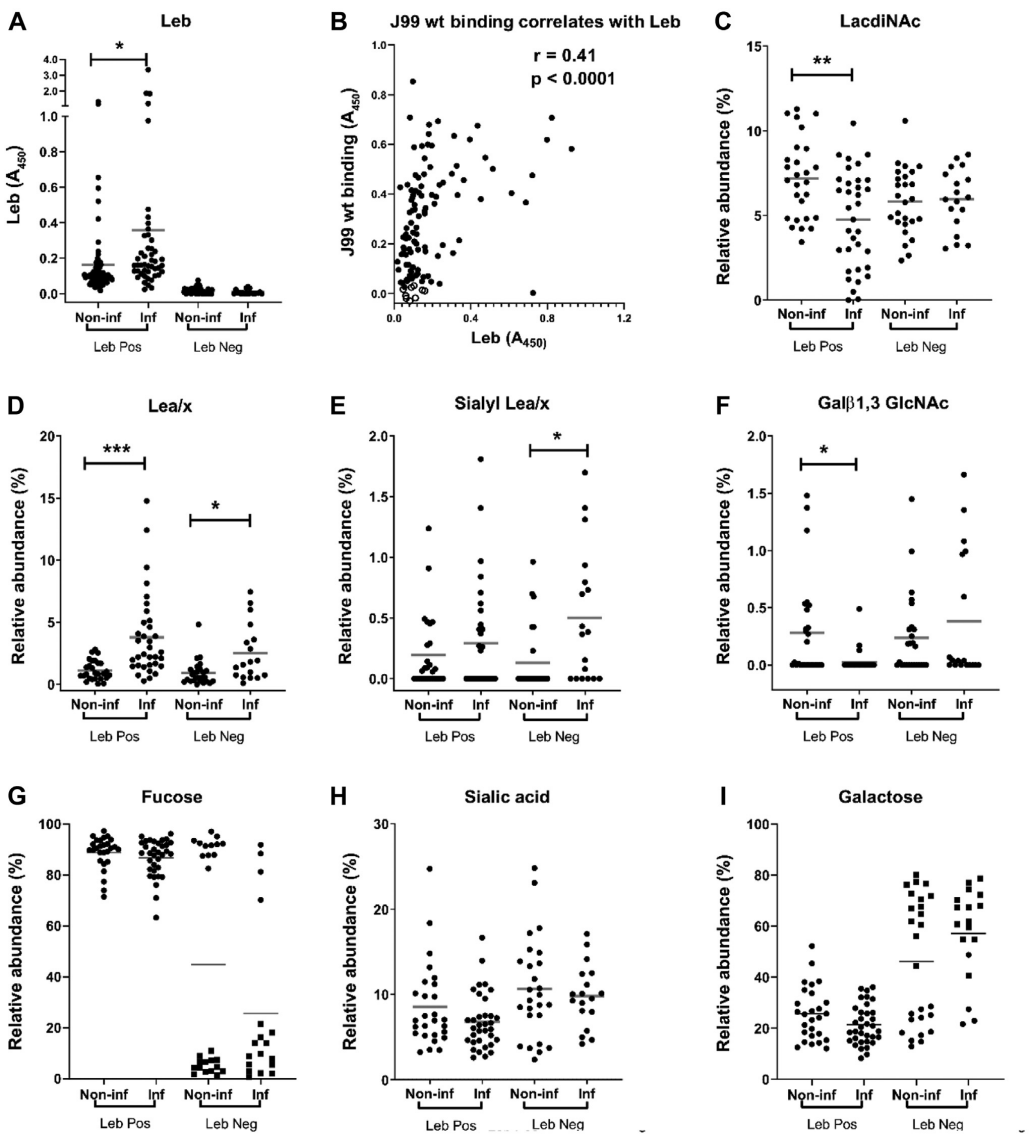
**Relative abundance (RA) of terminal glycan structures on gastric mucins from *Helicobacter* spp.-infected and -noninfected humans.** The datasets contain the RAs of the structures in all four mucin sources from each individual and have been grouped into Leb-positive (Leb Pos) and Leb-negative (Leb Neg) individuals either negative (Non-inf) or positive (Inf) for *Helicobacter* spp. *A*, the data points represent the Leb content of the mucins based on ELISA. *B*, J99 wt binding correlation with the amplitude of Leb in all four fractions from Leb-positive mucins. *White circles* denote mucins that did not bind statistically significantly better to J99 wt than wells with no mucins, and *black circles* denote mucins that bound to J99 wt. *C*–*I*, the data points represent the RA of glycan structures as determined by mass spectrometry (MS). In *G* and *I*, secretors are denoted with *circles* and nonsecretors as *squares*. Statistics. ∗*p* < 0.05, ∗∗*p* < 0.01, ∗∗∗*p* < 0.001, two-way ANOVA, the *bars* represent the mean. Leb, Lewis b.

*H. pylori* J99 wt binding to mucins correlated with the amplitude of the Leb signal (Fig. 7, *A* and *B*) and the gastritis score (Pearson’s *r* = 0.41; *p* < 0.0001 and *r* = 0.44; *p* < 0.001, respectively). Although Leb was associated with increased binding to *H. pylori* J99 wt, the relation was not linear, and binding also occurred to the majority of the Leb-negative samples (Fig. 6, *A* and *B* and supplemental Fig. S4, *A*–*C*), indicating additional epitopes involved in binding. Furthermore, among the Leb-positive samples, there were also a few Leb-positive mucins that did not bind to J99 wt (Fig. 6*B* and supplemental Fig. S4, *A*–*C*). This suggests that other factors, such as steric hindrance, may also limit binding even when Leb is present on mucins.

#### Fucosylation Rather Than Sialylation, LacdiNAc, and Type 1 LacNAcs Appear Responsible for the *H. pylori* Binding Differences Between Mucins From Infected and Noninfected Groups

To elucidate the structures responsible for binding and differences in binding between groups, we analyzed the carbohydrate structures present on mucins from infected and noninfected Leb-positive and Leb-negative individuals with focus on structures that *Helicobacter* spp. have been shown to bind to previously. *H. pylori* binding to mucins can occur to Leb and related fucosylated structures *via* BabA. There were no statistically significant differences in overall fucosylation between the groups, although among the Leb-negative mucins, there were two distinct populations with high *versus* low fucosylation (Fig. 7*G*). The samples with high fucosylation came from secretor individuals and the ones with low fucosylation from nonsecretor individuals, and there were fewer secretors among the Leb-negative samples from infected individuals than from the other three groups (Fig. 7*G*). The RA of Lea/x was higher among both Leb-positive and Leb-negative infected individuals (Fig. 7*D*). Leb was also increased among infected Leb-positive individuals compared with noninfected individuals (Fig. 7*A*). The increase in *H. pylori* binding among infected Leb-positive individuals could thus be driven by increased Leb levels compared with noninfected Leb-positive individuals. The cause of the decreased binding to mucins from infected compared with noninfected individuals among Leb-negative individuals is less clear, although there is a trend toward decreased fucosylation in the infected Leb-negative individuals compared with noninfected individuals, suggesting that fucosylation may play a role (Fig. 7*G*). Furthermore, the RA of fucose and α1,2-linked fucose did indeed seem to correlate better with J99 wt binding than Leb alone (Pearson’s *r* = 0.56 and 0.54 *versus* 0.41, *p* < 0.0001 for all three correlations), and the correlation between fucosylated structures and J99 wt binding remained when analyzing association among Leb-negative samples (*r* = 0.74, *p* < 0.0001). All except one of the individual structures that correlated with *r* above 0.4 with the amplitude of *H. pylori* J99 wt binding to human gastric mucins were fucosylated (supplemental Table S3).

J99 also carries the SabA adhesin. However, no differences in overall sialylation existed between the groups (Fig. 7*H*). Although differences were detected between the groups with regard to the RAs of sLea/x, the receptor for SabA, this was an increase in sLea/x abundance in the infected Leb-negative group (Fig. 7*E*), that is, the opposite pattern compared with J99 binding (Fig. 6*A*).

*H. pylori* has also been described to employ the LabA adhesin to bind to LacdiNAc, and J99 carries LabA. However, the LacdiNAc structure was decreased among Leb-positive infected mucins but not among Leb-negative infected mucins (Fig. 7*C*), suggesting that LacdiNAc is not responsible for the differences in binding between the groups.

*H. suis* was recently shown to bind to Galβ1,3GlcNAc, and this structure was decreased among pigs experimentally infected with *H. suis* (18, 25). However, although this structure was also decreased among infected Leb-positive humans, this was not the case among Leb-negative ones (Fig. 7*F*).

#### The Differences in *H. pylori* Binding Between Mucins From Infected *Versus* Noninfected Samples Are Dependent on BabA, Regardless of Leb Status

**Fig. 8:**
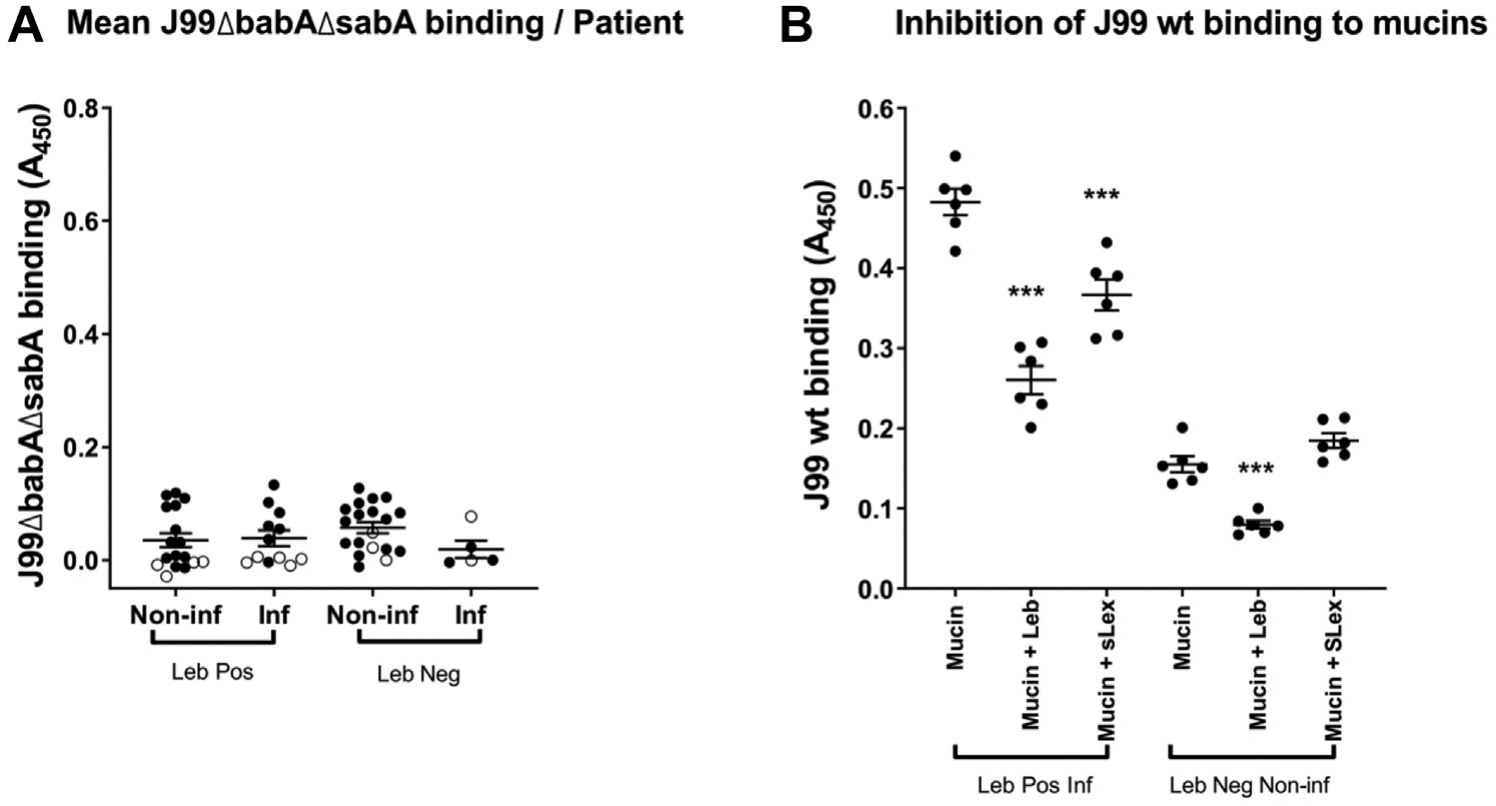
**Inhibition of *Helicobacter pylori* binding to Leb-positive and negative gastric mucins.***A*, binding of J99Δ*babA*Δ*sabA* to human gastric mucins isolated from noninfected (Non-inf) and *Helicobacter* spp.-infected (Inf) individuals. The data points represent the mean binding signal from the four mucin sources from each individual, *white circles* denote samples in which none of the four mucin types bound statistically significantly better to J99Δ*babA*Δ*sabA* than wells with no mucins and *black circles* denote mucins that bound J99Δ*babA*Δ*sabA*. The *bars* represent mean ± SEM. The scale is the same as for Figure 5 to allow comparison with the J99 wt. *B*, inhibition of binding using synthetic glycoconjugates. The six mucins with the strongest avidity for J99 wt within the Leb-positive infected and Leb-negative noninfected individuals were selected for the analysis. *H. pylori* J99 wt was pretreated with Leb-HSA or sLex-APD-HSA prior to addition to the mucins. Data are shown after subtracting the background control. The *bars* represent mean ± SEM, ∗∗∗*p* ≤ 0.001, one-way ANOVA, Dunnett's post hoc test. HAS, human serum albumin; Leb, Lewis b.

**Fig. 9:**
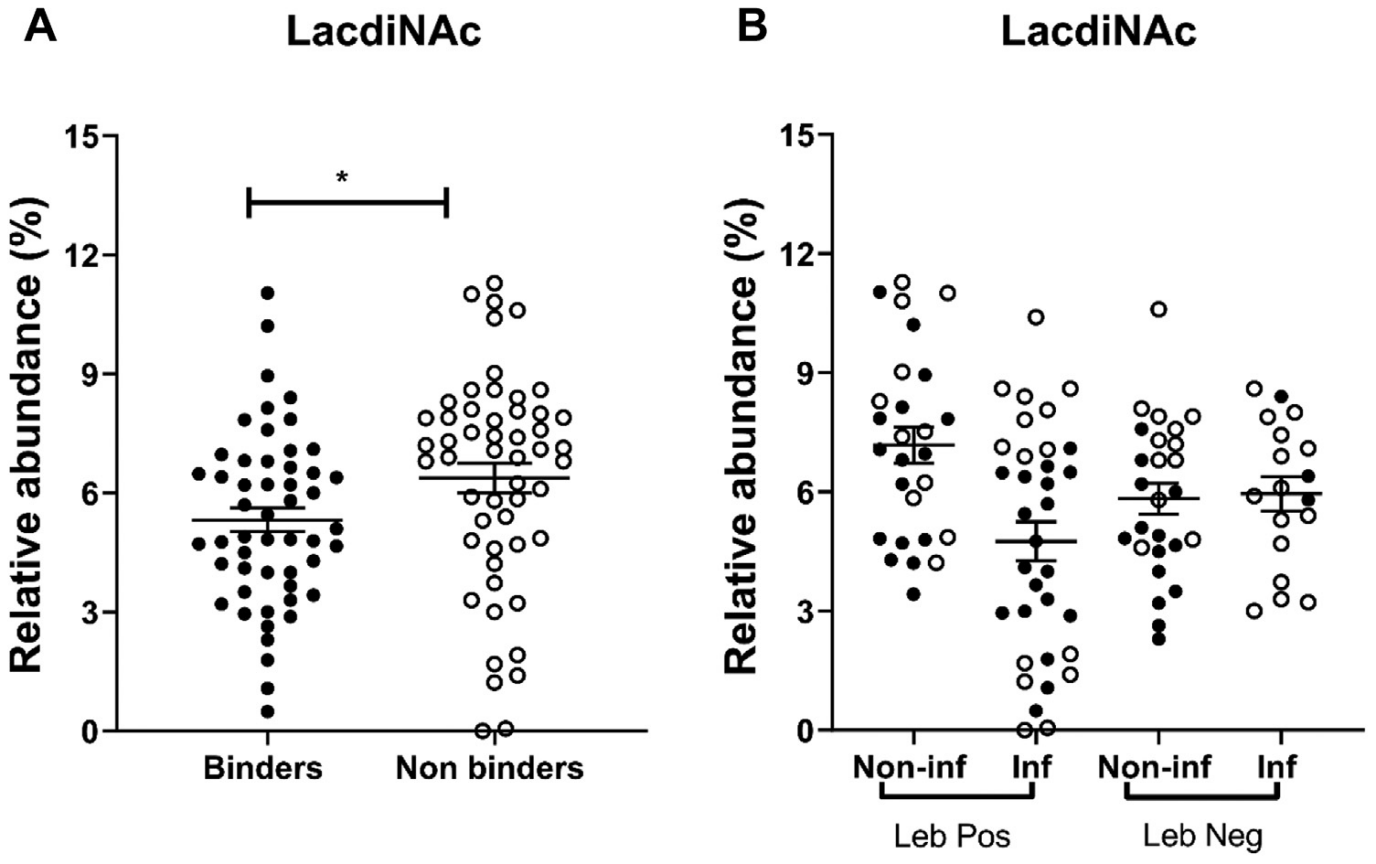
**LacdiNAc abundance of gastric mucins in relation to J99ΔbabAΔsabA binding.***A*, relative abundance of LacdiNAc among mucins that bound J99ΔbabAΔsabA (binders) and did not (nonbinders). ∗ indicates *p* ≤ 0.05, Student’s *t* test. *B*, lack of relation between binding of J99ΔbabAΔsabA to human gastric mucins isolated from *Helicobacter* spp.-infected (inf) and -noninfected (Non-inf) Leb-positive and -negative individuals. *Black circles* denote mucins that bound J99ΔbabAΔsabA, and *white circles* denote mucins that did not. The bars represent mean ± SEM.

J99 lacking the BabA and SabA adhesins (J99Δ*babA*Δ*sab*A) bound to 80% of the mucin samples, but the binding was low and did not differ with infection or Leb status (Fig. 8*A*). Binding of J99Δ*bab*AΔ*sabA* neither correlated with gastritis score (Pearson’s *r* = −0.21, *p* = 0.12) nor with the amplitude of the Leb signal (Pearson’s *r* = 0.03, *p* = 0.62). To investigate if LabA-dependent binding to LacdiNAc could be responsible for the low level of binding that remained after removing BabA and SabA, we analyzed the LacdiNAc content in relation to binding. However, the mucins that did not bind J99Δ*bab*AΔ*sabA* had a higher abundance of LacdiNAc than the mucins that bound J99Δ*babA*Δ*sabA* (*p* < 0.05), and no association was found between LacdiNAc abundance and binding within Leb-positive or Leb-negative samples, regardless of infection status (Fig. 9).

Binding of mucins with the highest *H. pylori* binding ability, that is, Leb-positive *Helicobacter* spp.-infected individuals and Leb-negative mucins from noninfected individuals to *H. pylori* J99 wt, was inhibited by Leb (Fig. 8*B*). Binding of mucins from Leb-positive *Helicobacter* spp.-infected individuals was partially inhibited by sLex-conjugates, whereas binding of Leb-negative mucins from noninfected individuals to J99 wt was not (Fig. 8*B*). *H. pylori* J99 wt binding ability to Leb-negative mucins significantly correlated with 35 structures whereof 24 fully characterized (Tables 5 and S4). Of these, 33 (94%) were fucosylated, and α1,2-linked fucose was a common theme among the fully characterized structures (Tables 5 and S4). In comparison, 285 of 423 (67%) of the structures present on Leb-negative mucins were fucosylated.

**Table 5:** Human mucin glycan structures whose RA correlated with a Pearson's *r* >0.4 with the amplitude of *H. pylori* J99 wt binding to human gastric mucins that were Leb negative based on ELISA results

Mass	Structures	Correlation of J99 wt binding with Leb-negative mucins			
		Pearson ρ	Adjusted *p* value	RA % (minimum–maximum)	SNFG
530	Fuc(α1–2)[GalNAc(α1–3)]Gal	0.69	<0.0001	0–5.52	
530	Fuc(α1–2)Gal(β1–3)GalNAc	0.61	0.0051	0–14.7	
733	Fuc(α1–2)[GalNAc(α1–3)]Gal(β1–3)GalNAc	0.72	<0.0001	0–7.2	
733	Fuc(α1–2)Gal(β1–3)[GlcNAc(β1–6)]GalNAc	0.76	<0.0001	0–4.7	
749	Gal(β1–4)GlcNAc(β1–3)Gal(β1–3)GalNAc	0.61	0.0049	0–2.3	
790	Gal(β1–4)GlcNAc(β1–3)[GlcNAc(β1–6)]GalNAc	0.55	0.0352	0–0.4	
821	Fuc(α1–2)Gal(β1–3)[Neu5Ac(α2-6)]GalNAc	0.72	<0.0001	0–0.4	
879	Fuc(α1–2)[GalNAc(α1–3)]Gal()[Fuc]GlcNAc	0.77	<0.0001	0–0.4	
895	GalNAc(α1–3)[Fuc(α1–2)]Gal(β1–4)GlcNAc(β1–3)Gal	0.78	<0.0001	0–3.4	
936	Fuc(α1–2)[GalNAc(α1–3)]Gal(β1–4)GlcNAc(β1–3)GalNAc	0.61	0.0041	0–3.1	
936	Fuc(aα1–2)[GalNAc(α1–3)]Gal(β1–3)[GlcNAc(β1–6)]GalNAc	0.82	<0.0001	0–2.1	
1098	Gal(β1–3)[Fuc(α1–2)[GalNAc(α1–3)]Gal(β1–4)GlcNAc(β1–6)] GalNAc	0.81	<0.0001	0–5.7	
1098	Fuc(α1–2)[GalNAc(α1–3)]Gal(β1–3)[Gal(β1–4)GlcNAc(β1–6)]GalNAc	0.74	<0.0001	0–13.04	
1098	Fuc(α1–2)Gal(β1–3)[GlcNAc(α1–4)Gal(β1–4)GlcNAc(β1–6)]GalNAc	0.68	0.0001	0–8.1	
1139	Fuc(α1–2)[GalNAc(α1–3)]Gal(β1–3)[GalNAc(β1–4)GlcNAc(β1–6)]GalNAc	0.81	<0.0001	0–3.3	
1244	GalNAc(α1–3)[Fuc(α1–2)]Gal(β1–4)GlcNAc[Fuc(α1–2)]Gal(β1–3)GalNAc	0.60	0.0068	0–0.7	
1244	GalNAc(α1–3)[Fuc(α1–2)]Gal(β1–3)[Fuc(α1–2)Gal(β1–4)GlcNAc(β1–6)]GalNAc	0.73	<0.0001	0–4.2	
1244	Fuc(α1–2)Gal(β1–3)[GalNAc(α1–3)[Fuc(α1–2)]Gal(β1–4)GlcNAc(β1–6)]GalNAc	0.72	<0.0001	0–18.6	
1301	GlcNAc(α1–4)Gal(β1–3)[GalNAc(α1–3)[Fuc(α1–2)]Gal(β1–4)GlcNAc(β1–6)]GalNAc	0.67	0.0002	0–2.1	
1447	GalNAc(α1–3)[Fuc(α1–2)]Gal(β1–3)[GalNAc(α1–3)[Fuc(α1–2)]Gal(β1–4)GlcNAc(β1–6)]GalNAc	0.79	<0.0001	0–10.7	
1504	Fuc(α1–2)[GalNAc(α1–3)]Gal(β1–4)GlcNAc(β1–3)Gal(β1–3) [GalNAc(β1–4)GlcNAc(β1–6)]GalNAc	0.68	0.0001	0–0.9	
1650	GalNAc(α1–3)[Fuc(α1–2)]Gal(β1–4)GlcNAc(β1–3) [GalNAc(α1–3)[Fuc(α1–2)]Gal()GlcNAc(β1–6)]GalNAc	0.68	0.0002	0–1.1	
1812	GalNAc(α1–3)[Fuc(α1–2)]Gal()GlcNAc(β1–3)[Fuc(α1–2)Gal(β1–4)GlcNAc(β1–3)Gal(β1–4)GlcNAc(β1–6)]GalNAc	0.75	<0.0001	0–1.7	
2177	GlcNAc(α1–4)Gal(β1–4)GlcNAc(β1–3)[GlcNAc(α1–4)Gal(β1–4)GlcNAc(β1–6)]Gal(β1–3)[Fuc(α1–2)Gal[Fuc]GlcNAc(β1–6)] GalNAc	0.71	<0.0001	0–1.4	

*p* Values are shown after adjusting for multiple comparisons using the Holm–Šídák method. Three of the glycans are denoted as peeling products, but they were included as they likely represent the terminal epitopes of larger structures.  = Fuc,  = Gal,  = GlcNAc,  = GalNAc, and  = NeuAc.

## Discussion

In the present study, a large diversity in the human gastric mucin *O*-glycome was identified on mucins from *Helicobacter* spp.-infected and noninfected stomachs. Both a larger number of unique glycan structures, a larger intraindividual diversity and larger interindividual variation were identified among *O*-glycans from infected stomachs compared with noninfected stomachs. *H. pylori* strain J99 bound to both Leb/H type 1 positive and negative mucins from individuals with and without *Helicobacter* spp. infection. Among Leb-positive samples, *H. pylori* binding was higher to mucins from *Helicobacter* spp.-infected individuals than to mucins from noninfected individuals. The differences in *H. pylori* binding ability among these four groups were governed by BabA-dependent binding to fucosylated structures. No effect relating to binding differences between groups was identified for other structures previously implicated in binding (*i.e.*, SLea/x, LacdiNAc, and Galβ3GlcNAc). Furthermore, we identified structures that correlated well with *H. pylori* binding and could be investigated further for their role in host–pathogen interactions.

The samples in the present study were selected to obtain four similarly sized groups of Leb-positive infected *versus* noninfected individuals and Leb-negative infected *versus* noninfected individuals, since Leb is a major epitope for *H. pylori* binding to gastric mucins (5, 42). Due to that Leb is present in the majority of individuals, we selected samples to obtain sufficient numbers of Leb-negative individuals to be able to study binding to structures other than Leb. We also excluded samples that were on the borderline between gastritis and no gastritis. Therefore, the overall glycan distribution does not proportionally represent the average population. In addition, the patient material was obtained from obese individuals since a large amount of material was needed for the analysis, which can be obtained from patients undergoing obesity surgery without creating risks for the patient. Other potential sources of material comes with other drawbacks: gastric juice contains a large proportion of salivary mucins, which are differently glycosylated and interacts with *H. pylori via* other structures than those present on gastric mucins (6), and material from stomachs affected by malignancy carry cancer-associated glycosylation changes (16). Over 40% of the US population is obese, so the studied population still represents a relatively large part of the population, and the infected group has a similar body mass index as the noninfected group. Furthermore, 10% of the patients in the study had diabetes, which is in line with that 10% of the US population has diabetes (43). We have not found any literature on mucin *O*-glycosylation changes in obesity patients, and we cannot make a fair direct comparison with the datasets produced previously by our group or other groups and our dataset, both because of that most of these datasets do not come from fully healthy individuals either, differences in methodology, and the number of structures identified in these studies is much lower per individual than in the current dataset. Serum protein *N*-glycosylation changes after severe calorie restriction in obese patients, most likely reflecting a decreased level of inflammation (44). However, the gastritis scores in the gastric samples used were in line with expected levels of gastritis from nonobese individuals, and we did not see signs of higher than normal sialylation or sulfation (glycosylation associated with gastritis), suggesting that the glycoprofiles presented here are similar to what could be expected in the general population, with the exception of that the proportion of Leb-negative individuals was higher.

The data presented here are consistent to what we previously published from gastric tissue (15). With only six of the 631 glycan structures being common to all 28 individuals in this study, we conclude that the *O*-glycan diversity among human gastric mucins is very high. This is in agreement with our previous study that identified 258 structures in ten samples from tumors, tumor adjacent, and healthy gastric mucosa. The data in the previous study (16) suggested that the *O*-glycan repertoires from healthy stomachs were relatively small and that the combination of blood groups and disease drive the diversity. The low number of structures identified among the three samples from healthy individuals in our previous study appears unusually low in comparison to that 60 to 180 structures were identified in each noninfected individual in the present study and a total of over 480 structures identified in this group. However, the principle that more structures and higher diversity were found among samples with gastric disease was confirmed in the current study too. Considering that the proportion of microbes is very low in these tissue samples (volume vice the mucus containing theaca in the human epithelial cells plus mucus is roughly 1000-fold higher than the microbial content), the increased number of structures is highly unlikely to be of microbial origin, as the abundances of such glycans most likely would be below the detection limit of the analysis. That the glycans presented are of human origin and not bacterial origin is further supported by that we did not identify glycans such as bacillosamine derivates previously shown to be present on *H. pylori* (45), in our samples. It is a bit surprising that Fucα1–2Galβ1–3(Fucα1–2Galβ1–4GlcNAcβ1–6) GalNAcol was present among the six structures found in all individuals, since nonsecretors do not carry the FUT2 enzyme. However, these epitopes are present at 100-fold lower levels than among the individuals that have a high abundance of this structure. Similarly, very low levels of glycans containing the A epitope were also found on glycans from some individuals that the hospital typed as being of blood group B or O. We speculate that the presence of such very low levels of these epitopes can be caused by promiscuity of other glycosyltransferases, as previous publications have shown that glycosyltransferases can both act on other substrates and add other monosaccharides than their main specificity, albeit with a lower efficiency (reviewed in Ref. (46)).

We further showed that glycans from *Helicobacter* spp.-infected individuals were larger than those from noninfected individuals. Although the present study was performed on samples from naturally infected individuals, entailing that cause *versus* effect is hard to decipher, increased glycan size has in a previous study been associated with decreased mucin production rate, possibly because of that the mucins stay longer time in the Golgi (47). Together with that *H. pylori* infection decrease the mucin production rate in the murine stomach (48), it is reasonable to believe that *H. pylori* infection in humans also may cause a decreased mucin production rate leading to increased glycan size. The larger interindividual variation found in the infected group coinciding with the increase in oligosaccharide size in this group reflected in an increased time for biosynthesis allowing small differences in glycosyltransferase activities between individuals to be exaggerated generating increased diversities of glycosylation profiles.

Immunohistochemistry has demonstrated an increase in the level of SLea/x in the gastric mucosa of naturally *H. pylori*-infected humans and in rhesus monkeys experimentally infected with *H. pylori*, and SLea was expressed in fewer surface mucous cells in the human stomach after *H. pylori* eradication compared with before eradication (7, 24, 49). In addition, previous MS studies have demonstrated that mucins from gastric tumors and intestinal metaplasia contained a higher proportion of negatively charged glycans, especially sulphated glycans compared with mucins from nonmalignant and noninfected stomachs (16, 17). In the current study, we found an increased abundance of SLea/x but no change in overall sialylation among infected samples, whereas the RA of sulphated structures was less than 1% in all groups. The limited difference in sialylation and sulfation between infected and noninfected individuals may be due to that there was no intestinal metaplasia or malignancy in the current material and also that at least in the rhesus monkey model of *H. pylori* infection, the increase in SLea/x levels is most pronounced early in infection (7). In order to compare the glycosylation profile between individuals, we used MS intensities to generate an overall picture of the glycosylation in each sample. This approach does not take into account differences in ionization efficiency between different glycans, that is, in negative ion mode, it is known that acidic glycans will be overrepresented. Thus, the low levels of acidic glycans found in the current study might be even lower than detected here. Using the MS approach comparing samples with similar types of glycans does however allow identification of glycan traits.

*H. pylori* binds to gastric mucins *via* several modes of adhesion: the BabA and SabA adhesins have been well characterized. Blood group A, B, H, and Leb antigens have been shown to interact with the *H. pylori* BabA adhesin (20, 50). and SabA recognizes the SLea and SLex antigens present not only on mucin *O*-glycans but also on glycolipids in the membrane of epithelial gastric cells (51, 52, 53). *H. pylori* has been described to carry a range of additional adhesins (54). Although we previously have detected binding to salivary mucins *via* some of these adhesins, we have detected no binding to gastric mucins *via* these adhesins (5, 6, 42). LabA has been suggested to bind to LacdiNAc on gastric mucins (21); however, although we recently confirmed that the J99 strain that we use in our laboratory carries LabA (23), we found no relation between binding and the presence of LacdiNAc on mucins even after removal of BabA and SabA. Lactotetraosylceramide has also been suggested to bind to *H. pylori* (55). The related bacterium, *H. suis*, was recently shown to bind to Galβ1,3GlcNAc, and this structure was decreased among mucins from experimentally infected pigs (18, 25). Similarly, among the Leb-positive human mucins used in the current study, Galβ1,3GlcNAc was decreased among mucins from *Helicobacter* spp.-infected individuals. However, among the Leb-negative mucins, there was no difference in Galβ1,3GlcNAc levels between mucins from infected *versus* noninfected individuals. Together, our results support that mucin glycans without the classical blood group H type 1, Leb and SLea/x can bind *H. pylori*. BabA-dependent binding to fucosylated structures dominate both the overall binding to gastric mucins and the differences in binding between the four patient groups. Of the structures identified to correlate well with *H. pylori* binding, most were fucosylated and included blood group–like epitopes. It would be relevant to investigate these structures and their role in host–pathogen interactions further.

Overall, *H. pylori* bound all four sources of mucins from all four patient groups. That there were mucins that did not bind *H. pylori* among the Leb-negative samples was not so surprising. However, a few samples in the Leb-positive groups did not bind to *H. pylori* J99, and one of the samples that had very high levels of Leb had very low levels of J99 binding. The BabA of strain J99 has a generalist binding phenotype and binds to Leb, H type 1, ALeb, and BLeb (20). Therefore, the presence of A or B epitopes did not explain the lack of binding, suggesting that other factors, such as steric hindrance, may also limit binding even when Leb is present on mucins. There appears to be 2 to 3 populations within the correlation between Leb and J99 binding levels. These populations may consist of a group with relatively low Leb levels but that may carry relatively large abundance of other J99 binding structures and other populations that are more directly dependent on Leb for adhesion.

In humans, Leb were expressed in fewer surface mucous cells after *H. pylori* eradication compared with before eradication (24). In line with these results, the human mucins positive for Leb from infected individuals showed increased binding to *H. pylori* compared with Leb-positive mucins from noninfected individuals in our study. Several studies suggest that mucins function as decoys for the adherence to membrane-bound glycoproteins and glycolipids, leading to decreased *H. pylori* density and gastritis (7, 8, 9, 56). Thus, increased binding capacity after infection appears beneficial to the host. The fact that expression of BabA and subsequent Leb binding is lost during early experimental infection in animal models (57) and mutations and/or recombination of BabA that lead to decreased binding to Leb occur in human patients during chronic infection (58) further supports this notion. A recent study by our group demonstrated that the *H. suis* binding ability of gastric mucins from *Helicobacter* spp.-infected human stomachs as well as experimentally infected pigs was lower than those from noninfected subjects (25). Together with decreased mucus production after *H. pylori* infection (48), a decreased ability of mucins from infected pigs and humans to bind *H. suis* implies that *Helicobacter* spp. infection inhibits binding and subsequent removal of the pathogen from the gastric niche. In the current study, human mucins negative for Leb from *Helicobacter* spp.-infected individuals showed decreased binding capacity to *H. pylori* compared with Leb-negative mucins from noninfected individuals. However, we cannot assume that this is due to an effect of infection, akin to the situation in *H. suis* infection, as this difference may be due to that there was a larger proportion of nonsecretors (*i.e.*, with lower level of fucosylation than secretors) in the infected group than the noninfected group.

In conclusion, we demonstrated that the gastric mucins have an enormous *O*-glycan diversity and that the diversity is partially driven by size, fucosylation, and infection. Given the importance of *H. pylori* translocation from gastric lumen to the epithelial barrier in shaping disease progression, understanding how specific glycan structures shape *H. pylori*–epithelial interactions during infection may aid in designing strategies to mitigate its pathogenic mechanisms. The absence of genetic information on glycosyltransferases such as FUT2 and FUT3 in patient samples is a limitation to the study. In other contexts, mucin glycans have been demonstrated to regulate pathogen phenotypes even at low concentrations, in a manner that likely is dependent on glycan complexity (59). The large and diverse gastric *O*-glycan structural library identified, including structures that correlate well with *H. pylori* binding, could be used to select glycodeterminants to experimentally investigate further for their importance in host–pathogen interactions and as candidates to develop glycan-based therapies.

## Data Availability

MS/MS data on the tentative structures are available at https://unicarb-dr.glycosmos.org/references/394. Raw data files are available at https://glycopost.glycosmos.org/entry/GPST000154.

## Supplemental data

This article contains supplemental data (16, 34, 35, 41).

## Conflict of interest

The authors declare no competing interests.
