# Supplementary material for: A Complex Connection Between the Diversity of Human Gastric Mucin O-Glycans, Helicobacter pylori Binding, Helicobacter Infection and Fucosylation
Source: Mol Cell Proteomics. 2022 Sep 29;21(11):100421. doi: 10.1016/j.mcpro.2022.100421 (PMC9661725; doi:10.1016/j.mcpro.2022.100421)
Supplement: Supplemental Figures S1–S4 and Tables S1, S3 and S4 [file mmc2.docx]

**A complex connection between the diversity of human gastric mucin *O*-glycans *Helicobacter pylori* binding, *Helicobacter* infection and fucosylation**

Gurdeep Chahal^1^, Médea Padra^1^, Mattias Erhardsson^1^, Chunsheng Jin^1^, Vignesh Venkatakrishnan^1^, Macarena Quintana-Hayashi^1^, János Tamás Padra^1^, Helen Stenbäck^1^, Anders Thorell^2^, Niclas G Karlsson^1^, Sara K Lindén^1^*

^1^University of Gothenburg, Department of Medical Biochemistry and Cell Biology, Gothenburg, 41390 Sweden

^2^Karolinska Institutet, Department of Clinical Science at Danderyds Hospital and Department of Surgery, Ersta Hospital, Stockholm, Sweden

*Corresponding author:

Prof. Sara K. Lindén, Department of Medical Biochemistry and Cell Biology, Institute of Biomedicine, Sahlgrenska Academy, University of Gothenburg, Box 435, 405 30 Gothenburg, Sweden;

E-mail: sara.linden@gu.se;

Phone: +46317863057,

Fax: +46317866330.

**Supplementary material**

**Figure S1. Representative images of Leb immunohistochemistry.** -------------------Page S-3

**Figure S2. Representative images of fluorescent in situ hybridization (FISH) for *Helicobacter* spp. and eubacteria on human gastric tissue sections.** -----------------Page S-4

**Figure S3. Distribution of human gastric mucin *O*-glycan structures among mucins from surface (n = 28) and gland (n = 28) mucosa.** ----------------------------------------------Page S-5

**Figure S4. Binding of *H. pylori* J99 wt to human gastric mucins isolated from *Helicobacter* spp. infected (Inf) and non-infected (Non-inf) individuals.** ----------------------------Page S-6

**Table S1. Immunoreactivity of isolated human gastric mucin samples.** ------------Page S-7

**Table S2.** **Glycan structures identified by mass spectrometry.** ----------------------- Excel file

Tables with all glycans, fully characterized glycans and the support for the identification of the individual glycans can be accessed by clicking the tabs at the bottom of the excel file.

**Table S3.** **Human mucin glycan structures whose relative abundance (RA) correlate with a Pearson`s r >0.4 with the amplitude of *H. pylori* J99 wt binding to human gastric mucins using all mucin samples.** ----------------------------------------------------------------------Page S-8

**Table S4. Human mucin glycan structures whose relative abundance (RA) strongly correlated (i.e. Pearson`s r = >0.4) with the amplitude of *H. pylori* J99 wt binding to human gastric mucins that were Leb negative. ------------------------------------------**Page S-9

**
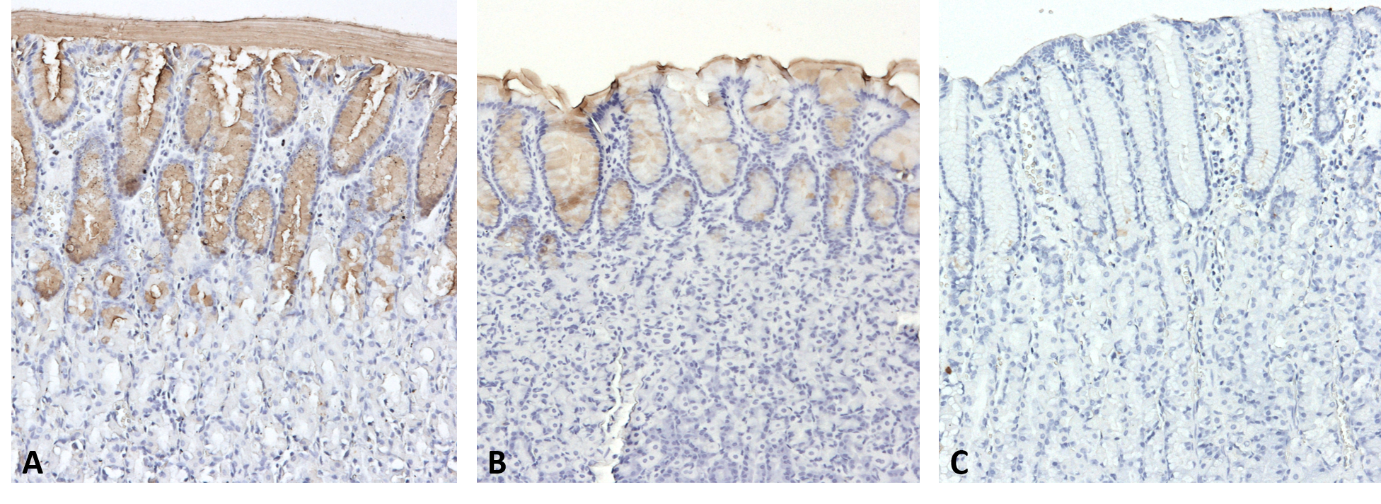
**

**Figure S1. Representative images of** Immunohistochemistry for Leb on human gastric tissue sections. The tissue sections were scored from 0 to 5 based on the intensity of the Leb antibody reaction (brown color). The representative images show an example for score 5 (A), score 2 (B) and score 0 (C).


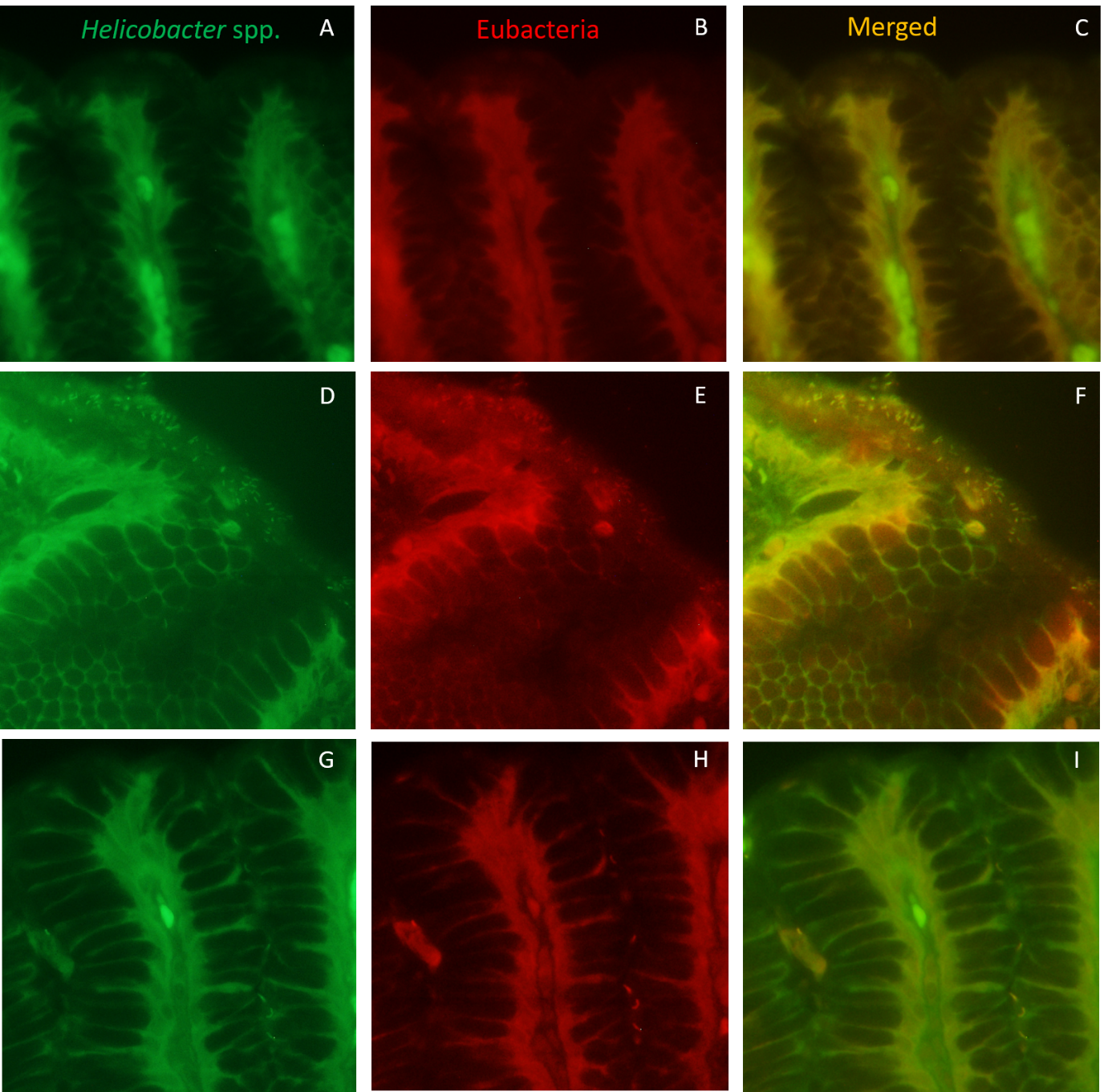
**Figure S2. Representative images of fluorescent *in situ* hybridization (FISH) for *Helicobacter* spp. and eubacteria on human gastric tissue sections.** **(A, D, G)** The *Helicobacter* spp. -specific probe gives a green signal where *Helicobacter* spp. are present. **(B, E, H)** The Eubacteria probe gives a red signal where any eubacteria are present. **(A, B, C)** Absence of bacteria on a human gastric tissue section confirmed to be *Helicobacter* spp. negative by PCR. **(D, E, F)** Presence of bacteria positive with both *Helicobacter* spp. and eubacterial probes in the surface mucus layer of the human stomach. **(G, H, I)** Presence of bacteria positive with both *Helicobacter* spp. and eubacterial probes in the gastric pits of human stomach. No bacteria were detected that were positive for the eubacterial probe but negative for the *Helicobacter* spp. specific probe in any sections.


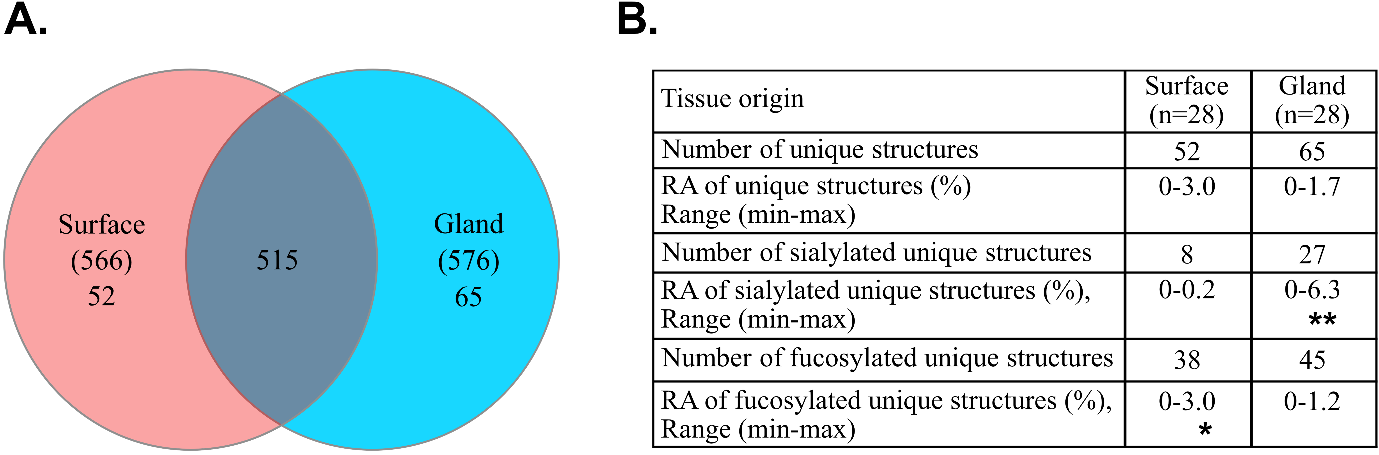


**Figure S3. Distribution of human gastric mucin *O*-glycan structures among mucins from surface (n = 28) and gland (n = 28) mucosa.** **(A)** Venn diagram. **(B)** Relative abundances (RA) of glycans. Stars indicate statistically significant difference in sialylated and fucosylated unique structures between groups, * and ** indicate p≤0.05 and p≤0.01, respectively, Mann- Whitney U test.

**
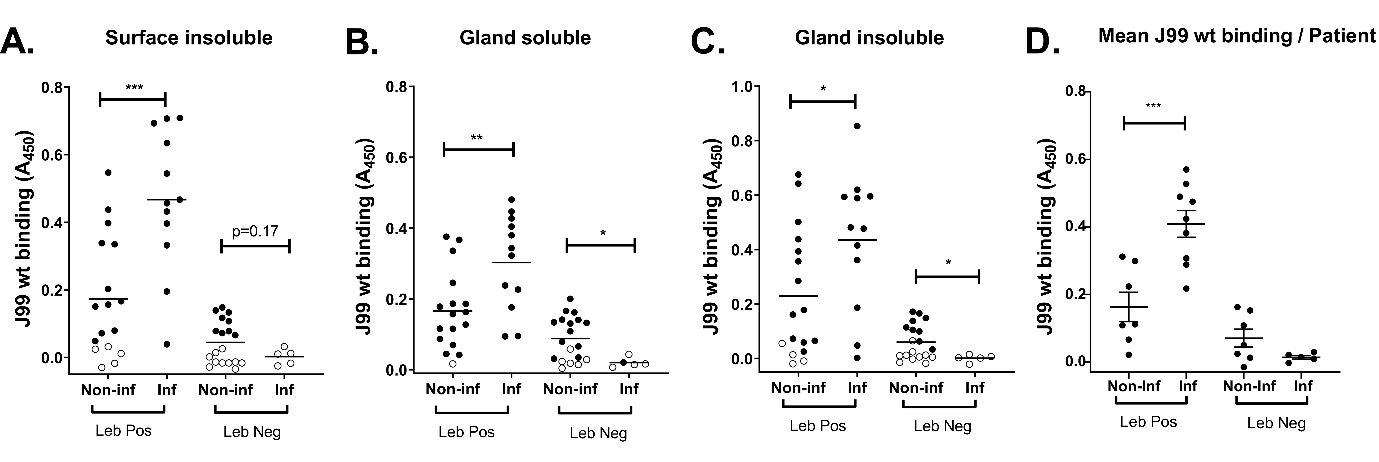
**

**Figure S4. Binding of *H. pylori* J99 wt to human gastric mucins isolated from *Helicobacter* spp. infected (Inf) and non-infected (Non-inf) individuals.** The binding signal to mucins surface insoluble **(A)** gland soluble **(B)** and gland insoluble **(C)** from each individual. White circles denote mucins that did not bind statistically significant better to J99 wt than wells with no mucins and black circles denote mucins that did. **(D)** The mean binding signal from the four mucin sources from each individual, only samples that were also analyzed with LC-MS included. The bars represent the mean, *p<0.05, **p<0.01, ***p<0.001; A-B: Student’s t-test, C: Mann-Whitney U test.

|  | Surface soluble (n=52) | Surface insoluble (n=52) | Gland soluble  (n=53) | Gland insoluble  (n=53) |
| --- | --- | --- | --- | --- |
| MUC5AC | 0.73 ± 0.04 | 0.54 ± 0.04 | 0.45 ± 0.03 | 0.45 ± 0.03 |
| MUC6 | 0.21 ± 0.02 | 0.18 ± 0.02 | 0.21 ± 0.02 | 0.19 ± 0.02 |
| Leb | 0.11 ± 0.02 | 0.15 ± 0.04 | 0.08 ± 0.02 | 0.13 ± 0.04 |

**Table S1. Immunoreactivity of isolated human gastric mucin samples.** The reactivity (A_450_) of antibodies against MUC5AC and MUC6 mucins and Leb in the isolated human gastric mucin samples was determined by ELISA. To compensate for differences in the level of mucin coated to the microliter plate, glycan detection assays were run in parallel to the ELISA for mucins and ELISA data were normalized to a glycan count of 20,000 (approximately 4 mg/mL mucin). The values represent mean ± SEM.

|  | Correlation with J99 WT binding | | | | |
| --- | --- | --- | --- | --- | --- |
| Mass | Structures | Pearson  ρ | Adjusted value | RA% (min-max) | SNFG |
| 628 | GalNAc(β1-4)GlcNAc(β1-3)GalNAc | 0.41 | 0.0104 | 0 - 0.7 | 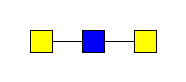 |
| 733 | Fuc(α1-2)Gal(β1-3)[GlcNAc(β1-6)]GalNAc | 0.63 | <0.0001 | 0 - 4.7 | 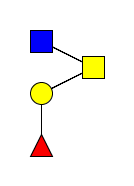 |
| 936 | Fuc(α1-2)[GalNAc(α1-3)]Gal(β1-4)GlcNAc(β1-3)GalNAc | 0.40 | 0.0153 | 0 - 3.1 | 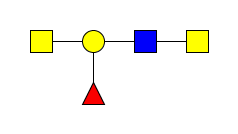 |
| 936 | GlcNAc(β1-3)[Fuc(α1-2)Gal(β1-4)GlcNAc(β1-6)]GalNAc | 0.40 | 0.0135 | 0 - 1.4 | 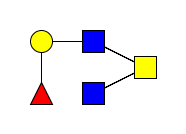 |
| 1041 | Fuc(α1-2)Gal(β1-4)[Fuc(α1-3)]GlcNAc(β1-3)Gal(β1-3)GalNAc | 0.43 | 0.0026 | 0 - 0.3 | 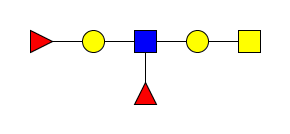 |
| 1041 | Gal[Fuc]GlcNAc-Gal(β1-3)GalNAc | 0.41 | 0.0068 | 0 - 0.3 | 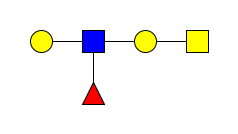 |
| 1139 | GlcNAc(β1-3)[GalNAc(a1-3)[Fuc(a1-2)]Gal(β1-3)GlcNAc(β1-6)] GalNAc | 0.40 | 0.0146 | 0 - 0.4 | 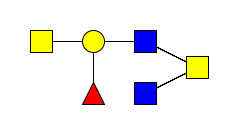 |
| 1186 | Fuc(α1-2)Gal(β1-3)[Neu5Ac(α2-3)Gal(β1-4)GlcNAc(β1-6)]GalNAc | 0.41 | 0.0104 | 0 - 2.5 | 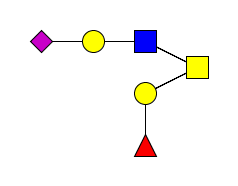 |
| 1609 | Fuc(α1-2)Gal-GlcNAc(β1-3)Gal(β1-3)[GalNAc(α1-3)[Fuc(α1-2)] Gal(β1-4)GlcNAc(β1-6)]GalNAc | 0.39 | 0.0236 | 0 - 0.7 | 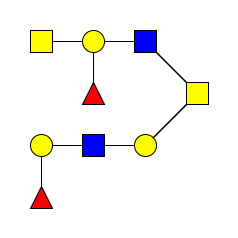 |
| 1771 | Fuc(α1-2)Gal(β1-4)GlcNAc(β1-3)[Fuc[Gal]GlcNAc(β1-6)]Gal(β1-3) [Gal(β1-4)GlcNAc(β1-6)]GalNAc | 0.39 | 0.0203 | 0 - 1.5 | 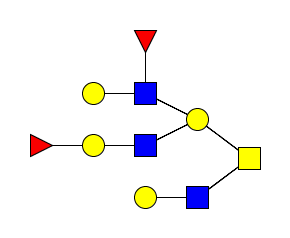 |
| 1812 | GalNAc(α1-3)[Fuc(α1-2)]Gal(β1-4)GlcNAc(β1-3)Gal(β1-3)  [GalNAc(α1-3)[Fuc(α1-2)]Gal(β1-4)GlcNAc(β1-6)]GalNAc | 0.40 | 0.0183 | 0 - 3.4 | 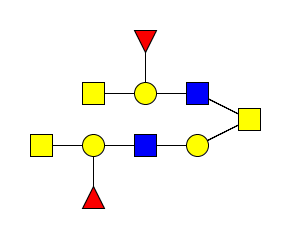 |

**Table S3.** Human mucin glycan structures whose relative abundance (RA) correlate with a Pearson`s r >0.4 with the amplitude of *H. pylori* J99 wt binding to human gastric mucins using all mucin samples. P values are shown after adjusting for multiple comparisons with the Holm-Šídák method.
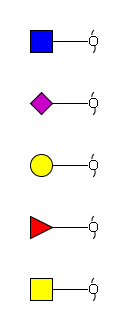
 = Fuc,
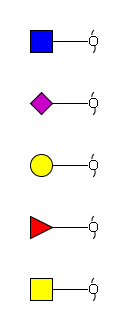
 = Gal,
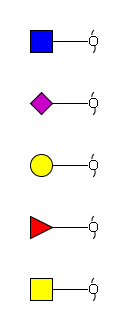
 = GlcNAc,
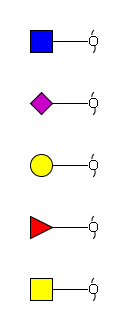
 = GalNAc and
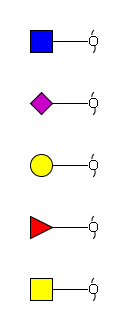
 = NeuAc.

| Mass | Composition | Putative structure | Hex | Hex  NAc | Fuc | Neu5Ac | Sulfate | Pearson  ρ | Adjusted p value | RA%  (min-max) |
| --- | --- | --- | --- | --- | --- | --- | --- | --- | --- | --- |
| 1609 | Hex3HexNAc4deHex2 |  | 3 | 4 | 2 | 0 | 0 | 0.77 | <0.0001 | 0 - 0.3 |
| 1609 | Hex3HexNAc4deHex2 |  | 3 | 4 | 2 | 0 | 0 | 0.77 | <0.0001 | 0 - 0.4 |
| 1609 | Hex3HexNAc4deHex2 |  | 3 | 4 | 2 | 0 | 0 | 0.63 | 0.0015 | 0 - 1.5 |
| 1609 | Hex3HexNAc4deHex2 |  | 3 | 4 | 2 | 0 | 0 | 0.54 | 0.0632 | 0 - 1.7 |
| 1666 | Hex3HexNAc5deHex1 |  | 3 | 5 | 1 | 0 | 0 | 0.64 | 0.0011 | 0 - 0.9 |
| 1812 | Hex3HexNAc5deHex2 |  | 3 | 5 | 2 | 0 | 0 | 0.74 | <0.0001 | 0 - 2.1 |
| 1828 | Hex4HexNAc5deHex1 |  | 4 | 5 | 1 | 0 | 0 | 0.76 | <0.0001 | 0 – 1.0 |
| 1958 | Hex3HexNAc5deHex3 |  | 3 | 5 | 3 | 0 | 0 | 0.56 | 0.0281 | 0 - 0.9 |
| 1974 | Hex4HexNAc5deHex2 |  | 4 | 5 | 2 | 0 | 0 | 0.55 | 0.0351 | 0 - 1.2 |
| 1974 | Hex4HexNAc5deHex2 |  | 4 | 5 | 2 | 0 | 0 | 0.72 | <0.0001 | 0 - 0.5 |
| 2542 | Hex5HexNAc7deHex2 |  | 5 | 7 | 2 | 0 | 0 | 0.63 | 0.0016 | 0 - 0.3 |

**Table S4.** Human mucin glycan structures whose relative abundance (RA) strongly correlated (i.e. Pearson`s r = >0.4) with the amplitude of *H. pylori* J99 wt binding to human gastric mucins that were Leb negative. P values are shown after adjusting for multiple comparisons using the Holm-Šídák method.
